# Proteomic analysis investigating kidney transplantation outcomes- a scoping review

**DOI:** 10.1186/s12882-023-03401-0

**Published:** 2023-11-22

**Authors:** Anna Rainey, Gareth J. McKay, Jane English, Ammarin Thakkinstian, Alexander Peter Maxwell, Michael Corr

**Affiliations:** 1 Centre for Public Health- Queen’s University Belfast, Belfast, UK; 2 Department of Anatomy and Neuroscience, University College Cork, Cork, Ireland; 3 Department of Clinical Epidemiology and Biostatistics, Faculty of Medicine Ramathibodi Hospital, Mahidol University, Bangkok, Thailand

**Keywords:** Kidney transplantation, Proteomic, Scoping review, Transplant outcomes

## Abstract

**Background:**

Kidney transplantation is the optimal treatment option for most patients with end-stage kidney disease given the significantly lower morbidity and mortality rates compared to remaining on dialysis. Rejection and graft failure remain common in transplant recipients with limited improvement in long-term transplant outcomes despite therapeutic advances. There is an unmet need in the development of non-invasive biomarkers that specifically monitor graft function and predict transplant pathologies that affect outcomes. Despite the potential of proteomic investigatory approaches, up to now, no candidate biomarkers of sufficient sensitivity or specificity have translated into clinical use. The aim of this review was to collate and summarise protein findings and protein pathways implicated in the literature to date, and potentially flag putative biomarkers worth validating in independent patient cohorts.

**Methods:**

This review followed the Joanna Briggs’ Institute Methodology for a scoping review. MedlineALL, Embase, Web of Science Core Collection, Scopus and Google Scholar databases were searched from inception until December 2022. Abstract and full text review were undertaken independently by two reviewers. Data was collated using a pre-designed data extraction tool.

**Results:**

One hundred one articles met the inclusion criteria. The majority were single-centre retrospective studies of small sample size. Mass spectrometry was the most used technique to evaluate differentially expressed proteins between diagnostic groups and studies identified various candidate biomarkers such as immune or structural proteins.

**Discussion:**

Putative immune or structural protein candidate biomarkers have been identified using proteomic techniques in multiple sample types including urine, serum and fluid used to perfuse donor kidneys. The most consistent findings implicated proteins associated with tubular dysfunction and immunological regulatory pathways such as leukocyte trafficking. However, clinical translation and adoption of candidate biomarkers is limited, and these will require comprehensive evaluation in larger prospective, multicentre trials.

**Supplementary Information:**

The online version contains supplementary material available at 10.1186/s12882-023-03401-0.

## Introduction

Kidney transplantation is the gold standard renal replacement therapy for most people living with end-stage kidney disease (ESKD) [1]. Transplantation has been shown to increase life expectancy by as much as 25 to 30 years compared to patients receiving dialysis and offers recipients a significantly higher quality of life [2]. Despite modern improvements in short-term kidney transplantation outcomes these have yet to translate to better long-term graft survival [3]. Transplant failure (graft loss) can be detrimental to overall health due to the need to return to dialysis treatment and the associated increase in morbidity and mortality [4]. Repeat transplantation is often challenging due to the development of anti-HLA antibodies limiting future donor options [5]. For wider society, transplant loss is associated with higher healthcare costs and increased demand for the limited number of available organs against a backdrop of increased ESKD prevalence [6, 7].

Despite the detrimental burden that kidney transplant failure places on both patients and society, early clinical biomarkers of sufficient sensitivity and specificity to detect failing grafts are lacking [8]. A kidney transplant biopsy is the most informative diagnostic tool but is an invasive procedure with potential complications including life-threatening bleeding [9]. Given the complication risk repeat or serial biopsies are rarely performed limiting its use in clinical practice to monitor or screen for graft dysfunction especially beyond the period immediately post-transplantation [10]. Other currently used non-invasive markers of graft dysfunction include proteinuria, serum creatinine, serum cystatin C, estimated glomerular filtration rate (eGFR), and for immunological graft dysfunction Donor Specific Antibodies (DSA) [11]. These non-invasive biomarkers are however limited by lack of specificity to an underlying disease process (with exception of DSA) and significant changes in these biomarkers are usually detected late in the disease process when clinical intervention is least effective [11].

Clinical proteomics studies can analyse a broad range of proteins from cells, tissues, and biofluids in a defined diseased state. By testing from minimally invasive biofluids (serum or urine), proteomic analysis has the potential to identify biomarkers of graft dysfunction that are more sensitive than currently used measures such as serum creatinine or proteinuria and offer improved accessibility over kidney transplant biopsy [12]. Analysis of samples prior to clinical presentation with graft dysfunction may also identify early biomarkers that are predictive of later graft loss, to facilitate early intervention and improve clinical outcomes [13].

Proteomic analyses costs have fallen considerably and modern analysis techniques, with high throughput mass spectrometry coupled with bioinformatic analyses, permit the quantification of thousands of proteins from a single sample [14]. Hence, it provides exciting opportunities to improve understanding of the biology of graft loss in kidney transplant recipients and potentially identify disease associated molecular pathways and novel diagnostic and/or prognostic biomarkers similar to other pathological conditions such as ovarian [15], prostate [16] and bladder cancer [17].

Despite recent advances in the technology, there is a considerable gap in our knowledge regarding protein signatures and molecular processes associated with kidney transplant outcomes. Systematic scoping review methodology was utilised given the variability of proteomic investigation techniques and diseases that affect graft function [18]. Therefore, this review was conducted to answer the following research questions: Which kidney transplant populations (including cohort size) have been studied in proteomic projects? What proteomic techniques and methodologies have been used and with what biological samples? What conditions known to affect kidney transplant outcomes have been studied using proteomic analysis? Is there any evidence on the diagnostic or prognostic value of proteomic evaluation of kidney transplant outcomes? Have specific proteins been identified as potential diagnostic or prognostic biomarkers?

Our aim was to complete a comprehensive summary of the current literature on proteomic profiles in kidney transplant recipients, collate proteomic findings to date and identify research gaps in this novel field which will inform future research.

## Materials and methods

### Study design and registration

This study review protocol was developed according to the methodological framework proposed by Arksey and O’Malley [19] and complies with the recommendations of the Joanna Briggs Institute for elaborating scoping reviews [18]. It has been registered with the Open Science Framework (DOI https://doi.org/10.17605/OSF.IO/JHP9Z). Reporting of the review was completed in accordance with the PRISMA extension for reporting scoping reviews [20]. Please refer to PRISMA checklist in Supplementary Table 1.

### Study definitions

Clinical proteomic analysis—analysis of the protein components expressed by a genome in its entirety [12]. Proteomic analysis includes different proteomes such as kidney tissue, urine, serum, and perfusion fluid.

Kidney transplant—allogenic human kidney transplants. This excludes what is known currently about xenotransplantation.

Outcomes – pathologies known to affect kidney transplant recipients and transplant graft success or failure.

### Search strategy

The search strategies for each database were designed collaboratively by the research team and with the assistance of a medical librarian. MEDLINE ALL, Embase, Web of Science Core Collection, Scopus and Google Scholar databases were searched. We conducted a grey literature search and reviewed studies for articles cited. Our search strategy combined Medical Subject Headings terms, their synonyms, and the Boolean operators “AND” and “OR”. An example of our MEDLINE search strategy can be viewed in Table 1*.* The search was carried out in December 2022 with no date limits applied and the results were not restricted to articles written in the English language. Citations identified in the search were compiled and exported to Endnote 20 (Clarivate Analytics, PA, USA) bibliographic software, and duplicates were removed.

**Table 1:** Medline search strategy

Search Terms	
1	exp proteomics/
2	Proteomic analy*.mp
3	Renal Transplant.mp. or kidney graft/
4	Renal transplant*.mp
5	Kidney transplant*.mp
6	Renal graft*.mp
7	kidney transplantation/ or Kidney graft*.mp
8	1 OR 2
9	3 OR 4 OR 5 OR 6 OR 7
10	8 AND 9

### Study selection

**Fig. 1:**
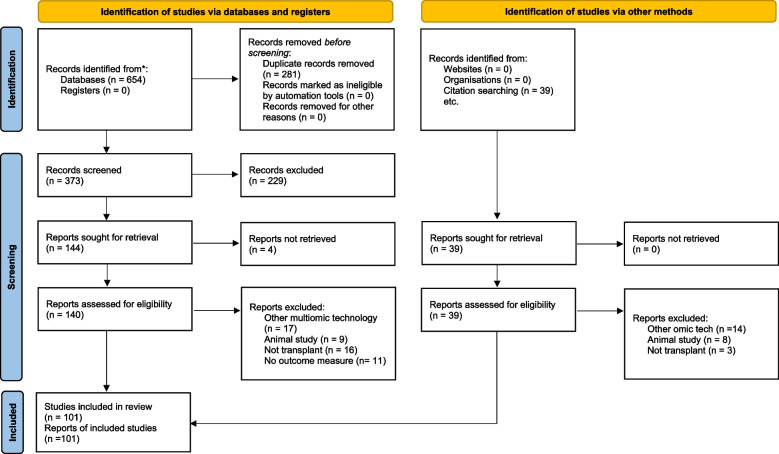
PRISMA flow diagram outlining the data collection process [21]. *Consider, if feasible to do so, reporting the number of records identified from each database or register searched (rather than the total number across all databases/registers).  **If automation tools were used, indicate how many records were excluded by a human and how many were excluded by automation tools.  From: Page MJ, McKenzie JE, Bossuyt PM, Boutron I, Hoffmann TC, Mulrow CD, et al. The PRISMA 2020 statement: an updated guideline for reporting systematic reviews. BMJ 2021;372:n71. doi: 10.1136/bmj.n71. For more information, visit: http://www.prisma-statement.org/

Following the removal of duplicate studies, title and abstract screening was performed independently by two members of the research team (AR & MC). Shortlisted studies underwent full-text review independently by both reviewers according to the inclusion and exclusion criteria outlined in Table 2*.* In the event of disagreement between both reviewers, a third reviewer (GMcK or APM) determined if the manuscript met the inclusion criteria. Each step of the study selection is detailed on a PRISMA flow chart with reasons for exclusions documented (Fig. 1).

**Table 2:** Inclusion and exclusion criteria

Review Category	Inclusion Criteria	Exclusion Criteria	Rationale
**Population**	Any human kidney transplant recipients	Animal studies, studies involving other solid organ transplants or bone marrow transplants	Due to studies aims and the varying biological processes and pathology in both animals and other human transplant populations
**Language**	Studies from all languages will be included	N/A	Studies written in languages other than research teams’ will be translated using available services at the research centre
**Time period**	From inception to present	N/A	Anticipation that most studies will be contemporary given nature of technology
**Study location**	All locations included	N/A	Worldwide problem not restricted to a geographical region
**Study Focus**	All studies which use any proteomic analysis technique to investigate kidney transplant graft outcome or pathology known to affect kidney transplant function; non exhaustive list rejection, viral infection, medication related injury, delayed graft function, acute kidney injury, prolonged ischaemia	Studies which do not specifically investigate prognostic and/or or diagnostic power of proteomics in kidney transplant grafts	Based on aim of the review
**Sample Type**	Any human biological sample including renal biopsy tissue, serum and urine from either donor or recipient, samples used during preparation of kidney transplant	N/A	In keeping with methodology broad range of samples relevant to kidney transplant will be included
**Proteomic techniques**	All studies profiling for differential protein expression changes etc. ELISA and western blot data (targeted towards specific candidates), and antibody-based multiplex immunoassays or mass spectrometry-based proteomics (discovery/untargeted proteomics)	N/A	Including targeted and untargeted proteomic techniques allows the review to capture full range of studies in kidney transplant recipients
**Types of Sources**	Articles, reviews, books, book chapters, text and opinion papers, web resources—grey literature	N/A	In keeping with scoping review methodology studies of all designs can be included the review including non-published material

### Data charting

Data charting was completed as a descriptive summary of key details of selected studies and their results. Data charting was piloted by two reviewers on 10 randomly selected articles and adjusted/corrected as required. The following data was extracted from the included articles; 1.) Year of publication 2.) Country/location of study 3.) Study design 4.) Study population 5.) Prognosis/Diagnosis studied 6.) Study sample (medium, storage) 7.) Proteomic analytical method 8.) Key results 9.) Individual proteins identified 10.) self-reported limitations of the studies. Data was extracted and included in tabular form using Microsoft Word. Disagreements between reviewers was mediated by third-party members of the research team. In the event of missing information authors of included studies were contacted for further details. Qualitative data synthesis was performed according to our review questions.

### Data summary

Our findings are presented in a narrative form and described according to the review questions. In keeping with scoping review methodology individual studies were not critically appraised or assessed for bias. Our findings provide an overview of the current literature whilst identifying potential knowledge gaps. As recommended by the Joanna Briggs Institute findings are presented to describe and map the current available evidence of proteomic analysis in kidney transplantation [18]. Reporting of the scoping review followed PRISMA-ScR guidance and include the reporting checklist.

### Changes to protocol

There were no changes to the protocol.

## Results

Database searching following duplicate removal identified 281 potential publications. Of these, 87 studies were included following independent abstract and full text review by two authors (AR & MC). The reasons for exclusion are outlined in the PRISMA flow diagram (Fig. 1) [21]. Primarily the reasons for exclusion were studies not specific to kidney transplant recipients (*n* = 16), studies that did not use proteomic techniques but other multiomic approaches (*n* = 17) and animal studies (*n* = 9). Upon further citation searching, 14 additional articles were included resulting in 101 articles for data extraction. A completed data extraction tool with study characteristics including population, disease studied and biomarkers of interest of all available studies can be seen in Supplementary Table 2.

### Study characteristics

Publications originated form 20 different countries, predominantly reporting studies of North American and European populations. The USA (*n* = 28) provided the highest number of publications, followed by Canada (*n* = 13) and China (*n* = 12). The earliest studies originated from 2003 and 2004 [22, 23].

A wide range of study designs were included such as exploratory, observational, case–control, cohort studies as well as other review articles. Notably, most were single-centre studies (*n* = 68) with relatively small sample sizes. There were only four multi-centre studies, including the two most recently published [24, 25]. Most studies investigated adult kidney transplant recipients, except five that focused on paediatric kidney transplant recipients [8, 26–29]. Three case–control studies used patients with stable graft function as control subjects [30–32]. Notably, Jacobs-Cacha et al. included chronic kidney disease patients as a further comparator for the proteins identified [33].

The biofluids and tissues used for proteomic profiling varied. Urine was most popular (*n* = 43) followed by serum (*n* = 19), urinary vesicles (*n* = 7), kidney transplant biopsy (*n* = 6) and perfusion fluid used on donor kidney prior to transplantation (*n* = 4). Three studies evaluated both serum and urine samples to compare proteomic profiles [33–35].

### Investigation techniques

Proteomic techniques used for evaluation in studies included different forms of Mass Spectrometry (MS) such as Liquid Chromatography (LC)-MS/MS, Surface Enhanced Laser Desorption Ionisation Time of Flight MS (SELDI-TOF MS) or Matrix-assisted laser desorption/ionisation-time of flight MS (MALDI-TOF MS). LC–MS/MS or shotgun proteomics is considered the gold standard investigation technique and advancements in this methodology has allowed for broader detection of multiple proteins that may be dysregulated in disease pathophysiology [36]. Less commonly used alternative techniques reported include large scale antibody arrays and proximity extension immunoassays, and ELISA. Most studies used multiple techniques as a process of internal validation of results.

### Outcomes of interest

The main outcomes included: interstitial fibrosis and tubular atrophy (IFTA); T-Cell mediated rejection (TMCR); antibody mediated rejection (AMR); acute tubular necrosis (ATN); delayed graft function (DGF) and calcineurin inhibitor toxicity (CNIT).

Acute rejection was the most studied outcome (*n* = 34 studies). Nine studies evaluated TCMR and four considered AMR; 21 studies failed to specify which acute rejection process they were investigating. Other studies did not investigate a specific contributory diagnosis to graft survival. For instance, Al-Nedwani et al. used LC–MS/MS to compare proteomic profiles in a case–control study design reporting variation between healthy controls, transplant recipients with good or poor graft function post-transplant [36]. The differential proteomic profiles identified candidate protein clusters including Vitamin D binding protein and Apolipoprotein E of potential diagnostic or prognostic value [36]. Several studies also compared multiple clinical outcomes. For example, Clotet-Frexias et al. used LC–MS/MS to differentiate biopsy proteomic profiles from transplant recipients with AMR (*n* = 7), TCMR (*n* = 11) and ATN (*n* = 12) [37]. They found proteins linked to metabolism, cytoskeletal and basement membrane functionality were heightened in those in the AMR group. Jacobs-Cacha et al. evaluated the proteomics in patients with known CNIT using ELISA to assess both urine and serum [33]. It was found that significantly higher urinary fascin-1 levels were correlated with biopsy proven CNIT compared to the control group of patients with chronic kidney disease [32]. Van Balkom and colleagues used multiplex immunoassays of perfusion fluid from deceased donor kidneys (*n* = 56) and found five differentially expressed proteins which were correlated with subsequent developed of DGF [38]. Sigdel et al. used LC–MS to investigate urinary proteomic profiles associated with IFTA and BK virus nephritis identifying 12 peptides upregulated in association with BK virus nephritis [39].

### Candidate proteins associated with kidney transplant

A wide range of proteins have been reported in association with kidney transplant outcomes representing a diverse biological profile. These ranged from immunoproteins such as chemokines and cytokines, to cytoskeleton structural proteins, and was dependent on the clinical sample profiled and proteomic technique employed. Whilst some studies focused on a single biomarker, others proposed panels of biomarker candidates. A full list of proteins reported in the included studies can be found in Supplementary Table 2*.* Highly cited proteins are discussed below.

### CXCL9 and CXCL10

CXCL9 and CXCL10 are chemokines and members of the CXCR3 family [26]. Both have well-characterised roles in leukocyte trafficking and immune function and are upregulated with acute rejection and may represent potential biomarkers for monitoring graft function [26, 40]. Blydt-Hansen et al. used ELISA to investigate biopsy-proven TCMR from urinary proteomic profiles of 51 paediatric kidney transplant recipients identifying an increased creatinine/CXCL10 ratio in both subclinical and clinical TCMR groups compared to non-TCMR patients [26]. Rotondi et al. conducted a case–control study that included 252 adult kidney transplant recipients dichotomising patient groups based on CXCL9 levels (greater or less than 272.1 pg/ml) before transplantation [41]. Serum ELISA assays identified higher pre-transplant serum CXCL9 in patients who subsequently developed graft failure [41]. Their study concluded that higher CXCL9 circulatory levels were indicative of graft failure [41]. Furthermore, a prospective multicentre observational trial of 280 transplant recipients that used both SELDI-TOF–MS and ELISA on urine samples, concluded that CXCL10 levels were similar between patients that experienced either TCMR or infection [29]. However, CXCL9 levels were higher in those with TCMR compared to infection suggesting lower urinary CXCL9 levels may potentially preclude TCMR as a diagnosis [29]. Ho et al., using both LC–MS/MS analysis and ELISA techniques, reported combined urinary metalloproteinase-7 MMP7 with urinary CXCL10 and creatinine improved differentiation of biopsy-proven IFTA from patients with normal histology [42].

### β2 microglobulin

Β2 microglobulin is a nucleated cell surface low molecular weight protein filtered by the glomerulus and used as a urinary biomarker of kidney damage [43]. Schaub et al. detected higher amounts of β2 microglobulin in the urine of patients with acute rejection using two different MS techniques [44]. Johnston et al. conducted a case–control study comparing the urinary profile of 34 transplant recipients with biopsy proven IFTA and 36 transplant recipients without. Using SELDI-TOF–MS and ELISA techniques, the study identified significantly higher levels of β2 microglobulin in the IFTA patient group [30]. Elevated β2 microglobulin was also reported in association with IFTA diagnosis by Kanzelmeyer et al. in a case–control study design that used capillary electrophoresis and MS techniques on urinary samples from 24 IFTA paediatric kidney transplant recipients and 36 control patients [27]. Furthermore, a cross-sectional study of urinary samples using SELDI-TOF MS and ELISA techniques identified a panel of proteins associated with IFTA that included β2 microglobulin [45].

### Neutrophil gelatinase-associated lipocalin (NGAL)

NGAL is a protein expressed in neutrophils and epithelium, including kidney tubules providing a range of physiological functions including a role in innate immunity and has been used as a urinary biomarker of acute kidney injury (AKI) [46, 47]. Heyne et al. used ELISA on urine samples (*n* = 182) to differentiate causes of AKI post-transplant with the highest urinary NGAL concentrations observed in those with biopsy proven TCMR [47]. Beyond this, MS and ELISA techniques on urine samples identified a panel of proteins that included NGAL to be more highly expressed in patients with biopsy proven IFTA [30]. Pianta et al. assessed the role of proteomics in predicting kidney graft function using ELISA techniques on urinary samples (*n* = 81) and identified NGAL as part of a panel of proteins that was predictive of delayed graft function [48].

### Urinary retinol-binding protein 4 (RBP4)

Urinary RBP4 is a lipocalin protein and a biomarker of tubular damage [49, 50]. Jeon et al. used LC–MS validated by ELISA on urinary samples from transplant recipients (*n* = 50) with different levels of graft function identifying significantly higher urinary RBP4 concentrations in patients with rapid transplant graft function decline compared to healthy controls with stable graft function supporting previous observations of increased RBP4 in patients with biopsy-proven IFTA in a paediatric population-based kidney transplant study [27, 49].

## Discussion

Proteomic evaluation to investigate kidney transplant outcomes offers the potential of clinically informative biomarkers of graft function. Molecular processes identified associated with graft outcomes varied based on outcome studied. Consistently graft dysfunction in studies appears to be associated with proteins found in molecular pathways involving comprehensive control networks for the actin cytoskeleton and renal tubular function. AMR was the most studied outcome and molecular pathways associated with it included complement activation, inflammatory cell activation and cell apoptosis although these findings warrant validation in independent patient cohorts. This scoping review considers the advances in the identification of novel structural and immune function proteins across a range of proteomic techniques. This review also considered the potential of the different proteomic approaches compared to the gold standard MS. However, despite technological advances, none of these biomarkers have been translated into clinical practice. A lack of standardisation across studies renders the need for validated multicentre data which will be required to enable the transition from a candidate protein to a clinically actionable biomarker. Other barriers have included the high cost associated with these approaches (though affordability in increasing with modern technological approaches) and the challenges associated with interpretation [51].

### Study design limitations

In keeping with scoping review methodology, the literature was not quality assessed but the preliminary research trials included in this review have many self-reported limitations. Firstly, a consistently reported limitation was the fact that most of the studies included were single-centre studies of relatively small sample size and the articles often indicated the need for further research using larger sample sizes, a more diverse sample population and multicentre data to validate their findings [51, 52]. Despite the increasing number of articles in the past 20 years reflecting an increased interest, very few of the candidate biomarkers have been followed up in multicentre trials. Secondly, the variability in sample collection and storage limit direct comparisons between studies [53, 54]. Differences between studies in terms of the choice of the sample substrate used also limits cross-study comparisons. Studies used urine, serum, perfusion fluid or biopsy samples and this added further variability between the quantification of proteins detectable. Thirdly, the variability in the volume and timing of urine collected and any potential contamination may further exacerbate the variability observed [55, 56]. Serum may represent a more stable substrate than urine, but it can be equally limited by the dynamic range of plasma proteins present, particularly when highly abundant proteins such as serum albumin mask the presence of leakage proteins or inflammatory cytokines present in relatively very low concentrations in comparison [57]. Fourthly, variation in the techniques for measuring differential protein expression, variation in sample preparation methods, and the inherent differences in sensitivity between techniques limits comparison of findings between studies. Another limitation identified within some of the preliminary studies was the lack of independent patient samples for validation for their main findings, and this is crucial for reproducibility and broader acceptance of the validity of proteomic assays [54].

### Limitations specific to transplantation

Most studies were limited by the lack of biopsy in the healthy control/stable transplant function groups. Given the inherent risks associated with this procedure, such as bleeding and infection, the absence of this biopsy data from healthy controls is understandable. However, graft rejection may be subclinical and therefore lack of biopsy data may overlook potential pathological changes which commonly precede clinical changes [58, 59]. Repeat biopsies on the same patient are usually not performed so data monitoring changes in the kidney graft is also lacking. In terms of the transplant outcome being considered, 21 of the studies examining acute rejection failed to specify whether this was TCMR or AMR. There are no candidate proteomic biomarkers to differentiate between these two distinct processes of acute rejection [59]. This is problematic, especially for risk stratification and prognosis, as AMR is more strongly associated with severe graft rejection [56, 59]. Furthermore, other conditions such as acute tubular injury or infection may co-exist alongside any rejection process [58]. Variation in the proteomic profiles of healthy controls can vary significantly from transplant recipients necessitating careful selection of appropriate comparators [60]. There is also significant heterogeneity between transplant recipients as they are subject to different underlying conditions, confounding factors or prior use of different immunosuppressive medications and this may limit the diagnostic potential of proteomic profiling techniques [60].

### Barriers to clinical translation

The potential proteomic biomarkers identified currently lack specificity and sensitivity to be clinically actionable. Although, many offer high negative predictive value, the positive predictive value is limited [51]. Many proteins upregulated in graft injury are commonly non-specific and may be upregulated in other conditions, such as urinary tract infections or other renal or urinary pathologies [61]. Kidney graft failure for both patients and clinicians is a precarious scenario with the higher risk of morbidity associated with a return to dialysis and the associated difficulties with re-transplantation [51, 62]. These high stakes mean that prognostic biomarkers for rejection would need to be incredibly sensitive and specific and so, despite the associated risks and limitations, these challenges to the gold-standard of kidney transplant biopsy remain [53].

This raises further questions regarding transplant outcomes/clinical conditions investigated by proteomic studies and whether the most appropriate clinical conditions are being selected to maximise translation into clinical practice. Most studies included in this review were focussed on these high-stake clinical events within the early post-transplantation period. Clinically this is a time of intense monitoring where outcomes in transplantation are excellent (even with complications) and invasive testing through transplant biopsy, is considered more acceptable [3, 9]. Arguably, studies focussing on longer term outcomes, have not seen similar improvements. There is an unmet need for useful clinical tests, such as those predicting graft outcome on borderline donor kidneys or long-term graft function after an initial stormy early post-transplantation course, and these types of tests may be more clinically valuable for quicker adoption into clinical practice [3, 4, 13, 30].

The use of urinary vesicles may also represent another barrier to clinical implementation. Urinary vesicles contain apoptotic bodies, microvesicles and exosomes which are involved in intercellular communication [63]. These components may more accurately reflect the true proteomic profile as they are secreted from a range of cells within the nephron including tubules and podocytes [24, 31, 64]. Nevertheless, specialised pre-processing to differentiate these vesicle-derived fractions may be less amenable to adaptation for clinical practice and so results based on urinary vesicles are less actionable.

Modern high throughput mass spectrometry coupled with bioinformatic analyses allows researchers to analyse thousands of proteins from a single sample [14, 55]. However, these technologies can identify a large number of proteins where the biological role of potential biomarkers is unclear or even questionable. This can limit clinical translation especially when candidate biomarkers such as CXCL9 and CXCL10 are identified in a wide range of pathologies such as acute rejection, infection, ATN and IFTA [26, 29, 41, 42]. Proteomic pathway analysis to better understand the functional role of detected proteins through complex interactions of molecular disease pathways was underutilised by studies included in this review [65]. Collaborative working between researchers to develop protein pathway databases in kidney transplantation, like those explored in oncology, would significantly help in developing hypothesis driven proteomic research [66, 67]. This would allow targeted investigation of potential biomarkers or panels for specific disease states linked to the underlying disease processes and pathophysiology and enhance potential translation of research findings into clinical practice.

### Future directions

This scoping review highlights the complexity of kidney transplant processes associated with graft failure and why it is likely to necessitate a panel of biomarkers rather than a single protein to prove useful [65]. This means a move towards broader and non-targeted assays that have the capability to simultaneously examine multiple candidate proteins [68]. Current technological advances now facilitate this through the development of protein biomarker platforms. Such panels can distinguish molecular signatures and enable simultaneous measurement of a large number of proteins with previously reported success in the renal field [55, 69, 70]. In one trial assessing kidney function decline over five years, up to 80 candidate proteins were investigated, of which 28 were significantly associated with a decrease in annual eGFR [71]. Another study examined albuminuria as an independent cardiovascular risk factor and, using proximity extension assay techniques, investigated 92 potential protein biomarkers [72]. It identified five cardiovascular proteins that were significantly associated with albuminuria. Whilst identifying a panel of proteins may be the next step forward, there is a need for quantification if these markers are to be become clinically informative [55, 56].

The major barrier to clinical utilisation of biomarkers, including those highlighted in this review, is the failure to move beyond discovery phase studies [73]. Validation studies are required to progress the field. Increased collaboration between research teams and the formation of consortia/biobanks could address the issue of low cohort sizes that limit proteomic transplantation studies. Data sharing and proteomic pathway analysis will help map the proteome in kidney transplant recipients in health to allow better understanding of variations in different pathologies.

Clinically actionable proteomic evaluation beyond kidney transplantation has seen progress in other fields of medicine, such as oncology. These approaches have been investigated as a screening tool for ovarian cancer staging [64]. Proteomics has also improved molecular understanding through the identification of several urine biomarkers for non-muscle invasive bladder cancer [74, 75]. The technology has also shown promise in facilitating personalised treatment options for prostate cancer patients [76]. In terms of evaluating the impact of immunosuppression on proteomic profiles, other specialties that have considered this issue include rheumatology. For example, protein extension assay techniques evaluated 92 plasma inflammation-related proteins in relation to Tofacitinib treatment for Rheumatoid Arthritis, its effect on cytokine expression and the possibility of predicting treatment response [77].

## Conclusion

Whilst there is a need for non-invasive biomarkers to monitor kidney graft function, traditional proteomic approaches have not yet delivered robust improvements to clinical practice. The studies included in this review identify the limitations of mainstream approaches and study design. Prospective, large-scale, and multicentre trials with validation sets are the next phase in the application of proteomics to kidney transplant medicine. There is a need to improve the detection of early rejection and subsequent higher risk of graft failure. If proteomic profiles could reliably discriminate between transplant recipients with and without early rejection, then immunosuppressive regimens could be modified for those transplant recipients at highest risk of rejection. The recent technological advancements in proteomics which have improved sensitivity and throughput mean thousands of potential candidate biomarkers can be evaluated. As this technology advances further, there will be more opportunities and greater potential for the biomarkers identified to be validated in independent cohorts, clinically trialled for diagnostic accuracy, and for translation into clinical practice. If successful in this transition, non-invasive proteomic biomarkers could revolutionise the way in which kidney grafts are monitored for both clinicians and patients, and significantly improve clinical outcomes.

### Supplementary Information

**Additional file 1.** Preferred Reporting Items for Systematic reviews and Meta-Analyses extension for Scoping Reviews (PRISMA-ScR) Checklist.

**Additional file 2. **Complete chart of included studies and proteins of interest.

## Data Availability

All relevant data and materials is included in the manuscript and supplementary materials.

## Abbreviations

AKI: Acute kidney injury
AMR: Antibody mediated rejection
ATN: Acute tubular necrosis
CNIT: Calcineurin inhibitor toxicity
DGF: Delayed graft function
DSA: Donor specific antibodies
eGFR: Estimated glomerular filtration rate
ESKD: End-stage kidney disease
IFTA: Interstitial fibrosis and tubular atrophy
LC–MS/MS: Liquid Chromatography-MS/MS
MALDI-TOF MS: Matrix-assisted laser desorption/ionisation-time of flight MS
MS: Mass Spectrometry
SELDI-TOF MS: Surface Enhanced Laser Desorption Ionisation Time of Flight MS
TCMR: T-cell mediated rejection
