# Supplementary material for: Proteomic analysis investigating kidney transplantation outcomes- a scoping review
Source: BMC Nephrol. 2023 Nov 22;24:346. doi: 10.1186/s12882-023-03401-0 (PMC10666386; doi:10.1186/s12882-023-03401-0)
Supplement: Supplementary file 2 — Additional file 2. Complete chart of included studies and proteins of interest. [file 12882_2023_3401_MOESM2_ESM.docx]

| **Author**  **(Year of publication)** | **Study Type** | **Population** | **Outcome studied** | **Sample Type** | **Proteomic technique used** | **Key findings/ Biomarkers of interest** | **Self-reported limitations** |
| --- | --- | --- | --- | --- | --- | --- | --- |
| Al-Nedawi (2019)  Canada [1] | Case control study | Group 1: 10 healthy volunteers, 20 kidney transplant patients divided equally.  Between good and poor prognosis based on slope of eGFR. Group 2  6 transplant patients (3 with stable and 3 with rapidly declining GFR), 3 healthy controls | Comparison of proteome and transplant outcome. No specific diagnosis. | Macrovesicles from plasma | Tandem Mass Spectrometry MS/MS sequencing | 8 clusters of proteins which may have diagnostic / prognostic factor for transplant outcome based on variation seen between groups e.g. Vitamin D binding protein and Apolipoprotein E. Group 2: 5 clusters of differentially expressed proteins. These clusters could discriminate between each group of the patients and the healthy subjects, and distinguish between the two groups of patients to reflect the outcomes of kidney transplants. | Preliminary Data, small patient groups. |
| Argani (2020)  Iran [2] | Review study |  |  |  |  | Most new biomarkers have high negative predictive value for rejection but not enough positive predictive value to allow a treatment decision for patients before renal biopsy. Dedicated, prospective, interventional trials are required to demonstrate that the use of these biomarkers improves patient or transplant outcomes. |  |
| Bañón-Maneus (2010)  Spain/Australia [3] | Prospective cohort study | 36 KTR. 12 with no IFTA, 12 with mild IFTA and 12 with severe IFTA | Stage of IFTA | Morning spot urine samples | MALDI-TOF-MS, MS/MS sequencing | 14 peptides were increased in groups with IFTA in comparison with the IFTA-I or IFTA-0 groups and seven were decreased | Larger and more diverse patient population required |
| Blydt-Hansen (2015) Canada [4] | Cohort Study | 51 Paediatric Kidney transplant recipients | Detection of acute T cell mediated rejection (TMCR) | Urine | ELISA | urinary CXCL10: creatinine (Cr) was significantly increased in both subclinical (4.4 ng/mmol, P < 0.001) and clinical TCMR (24.3 ng/mmol, P < 0.001) compared to noninflamed histology (1.4 ng/mmol, normal histology and IFTA). | -reliance on histology for classification  - repeated sampling in some patients  - findings need to be confirmed in a large, unselected population of paediatric renal transplant recipients |
| Bohra (2013)  USA [5] | Review Study |  |  |  |  | Future studies should be focussed on kidney transplant biomarker discovery 1.) goal-combinational markers 2.) longitudinal study design 3.) Ensuring sample quality and integrity 4.) Key focus on potential translation to clinical practice |  |
| Braun (2019)  Germany [6] | Prospective cohort study | 22 living donor KTR. 22 independent validation cohort. | Graft survival- no specific condition | Urinary extracellular vesicles | Western Blot, Untargeted. Mass Spectrometry.  Targeted LC-MS parallel reaction monitoring. | Small urinary extracellular vesicle proteome varied during transplantation. Complement activation was among the most dynamically regulated components. a correlative analysis identified putative prognostic markers of future allograft function. One of these markers –phosphoenol pyruvate carboxykinase (PCK2) – could be confirmed using targeted MS in an independent validation cohort of 22 additional patients | No gold standard in separation of vesicle protocol limiting interpretability. Small homogenous cohort. |
| Brouard (2008)  France [7] | Meeting Report |  |  |  |  | 2 strategies: 1) a profiling strategy that can provide a global non-identified description of the disease (SELDI-TOF, and (2) a global and “shotgun” identification (nano-LCMS/MS). The reliability of these technologies depends on (1) the clinical/biologic design  of the study and the statistical analysis (2) the sample management (3) the low concentration of the proteins. To date, only five biomarkers have been validated in the field of oncology, probably because of the magnitude of these obstacles |  |
| Cassidy (2015)  Ireland [8] | Case control | 34 KTR patients with biopsy proven IFTA. Control group of 36 KTR 1 year post-transplant with stable renal function | IFTA | Midstream Urine samples | Mass Spectrometry for detection. Protein quantification was performed using enzyme immunoassay test kit (ELISA) | β2 microglobulin, neutrophil gelatinase-  associated lipocalin, clusterin, and kidney injury biomarker 1 were expressed at significantly higher urinary concentrations in patients with IFTA compared to those with normal kidney function. | Small sample numbers |
| Carreras‑Planella (2020)  Spain [9] | Observational study | 23 KTR with TCMR, CNIT and normal kidney function. Further 41 KTR patients recruited as independent validation cohort. | Fibrosis in graft. TCMR and CNIT. | Urinary extracellular vesicles pre biopsy | Discover phase using shotgun mass spectrometry. Targeted proteomics and ELISA in validation. | Differential expression of vitronectin  (VTN) in patients displaying chronic interstitial and tubular lesions (>2 according to Banff criteria) | Limited number of patients in ELISA cohort. Biopsies performed prior to most recent Banff classification |
| Carreras‑Planella (2020)  Spain [10] | Observational pilot study | 17 KTR (7 normal kidney function, 5 CNIT, 5 IFTA) | CNIT vs IFTA | Urinary extracellular vesicles | Mass spectrometry | Plakin and Uroplakin protein families were upregulated in the CNIT cohort. | Pilot Study |
| Chakraborty (2018) USA [11] | Review Article |  |  |  |  | Acute rejection may represent a more inflammatory process than chronic rejection. Proteomic approaches may offer a better understanding of this inflammatory pathophysiology. |  |
| Chowdhury (2013)  UK [12] | Review Article |  |  |  |  | Development of protein MS has permitted the profiling of low-risk samples such as blood and serum on a much larger scale  There is still a need for clinical trials to be biomarker-led. |  |
| Christians (2016)  USA [13] | Review Article |  |  |  |  | Major bottleneck if moving beyond biomarker discovery stage and moving towards clinical validation with larger cohorts |  |
| Cibrik (2013) USA [14] | Case Control | Group 1 – Healthy controls n=10  Group 2 – Stable patients post-transplant n=19  Group 3 – Rejecting patients n=15 | Detection of biopsy-proven acute rejection | Serum | ELISA | Identified 10 serum proteins from 19 renal transplant patients with stable renal function, which are differentially expressed, compared to healthy control subjects.  identified 17 proteins that differentiate rejecting renal transplant recipients from stable renal transplant  Of these 17 proteins, nine have lower levels expression and eight have higher levels of expression in the rejecting cohort as compared to the stable renal transplant cohort. GF, GM-CSF,IL-1R1, KIM-1, MCP-1, and MIF associated with rejection.  Results support that a specific pattern of protein expression or “protein signature” may be able to differentiate between stable transplant patients from those with rejection | - lack of protocol renal transplant biopsies in the stable renal transplant cohort  - small sample size |
| Clarke (2003)  USA [15] | Observational Study | Thirty-four from 32 KTR at various stages post transplantation.  17 with acute rejection and 15 patients with no rejection. Those <4 days post-transplant were excluded due to high inflammatory state. | Acute transplant rejection | Urine | SELDI mass  spectrometry | The best candidate biomarkers demonstrated highly successful diagnostic performance: 6.5 kDa (AUC0.839,  P.0001), 6.7 kDa (AUC 0.839, P.0001), 6.6 kd (AUC 0.807, P.0001), 7.1 kDa (AUC0.807, P.0001), and 13.4 kDa (AUC0.804, P.0001). | Large numbers of peaks identified with variation between samples of same clinical group. |
| Clotet-Freixas (2020)  Canada [16] | Case control study | 30 transplant biopsy with AMR, TMCR and ATN (taken for clinical reason) | Differentiation between TMCR, AMR and ATN | Kidney Transplant Biopsy | Tandem MS | A total of 107 of 2026 glomerular and 112 of 2399 tubulointerstitial proteins was significantly differentially expressed in AMR versus TMCR; 112 of 2026 glomerular and 181 of 2399 tubulointerstitial proteins were significantly dysregulated in AMR versus ATN (P<0.05). Glomerular and tubulointerstitial laminin subunit *γ*-1 (LAMC1) expression decreased in AMR, as did glomerular nephrin (NPHS1) and receptor-type tyrosine-phosphatase O (PTPRO). Galectin-1 and cathepsin-V were upregulated in AMR. | Small sample focussed on extreme phenotypes |
| Cohen-Freue (2010)  Canada [17] | Case-control discovery study | 32 KTR (11 with TCMR, 21 with no TMCR) | Detection of biopsy proven TCMR | Serum | Isobaric tagging for relative and absolute protein  quantification (iTRAQ). Results validated with ELISA. | 18 plasma proteins that encompassed processes related to inflammation, complement activation, blood coagulation, and wound repair exhibited significantly different relative concentrations between patient cohorts (p value <0.05). Twelve proteins  with a fold-change >1.15 were selected for diagnostic purposes: even were increased (titin, lipopolysaccharide-binding protein, peptidase inhibitor 16, complement factor D, mannose-binding lectin, protein Z-dependent protease and β_2_-microglobulin) and five were decreased (kininogen-1, afamin, serine protease inhibitor, phosphatidylcholine-sterol acyltransferase, and sex hormone-binding globulin) | Precise clinical phenotypes with clear diagnoses |
| Gao (2009)  China [18] | Observational Study | 12 samples used for 2-D DIGE and MS  Analysis; 4 KTR  AR, 4 KTR with stable), and 4 normal volunteers. | Detection of biopsy proven acute graft rejection | Serum | Two-dimensional  differential in-gel electrophoresis (2-D DIGE) and reversed phase high-performance liquid chromatography (RP-HPLC) followed by electrospray ionization mass spectrometry (ESI-MS) | 22 differentially expressed proteins were identified in serum from AR group. These proteins included complement C9 precursor, apolipoprotein A-  IV precursor, vitamin D-binding protein precursor, beta-2-glycoprotein 1 precursor, etc. Vitamin D-binding protein was the only confirmed by ELISA in the independent set of serum samples. | Many proteins unable to be confirmed. |
| Gunther (2014)  Canada [19] | Case control Study | KTR 20 with AR episode within 30 days of transplantation, 20 with no AR within 6 months of transplantation. | Detection of acute rejection | Serum | iTRAQ MALDI-TOF/TOF  Mass Spectrometry | No variation with proteomic analysis alone between two groups. Required combination with genomic data for sufficient accuracy in determination between AR and no AR. | Lack of validation results |
| Gwinner (2016)  Germany [20] | Review Article |  |  |  |  | Recommendations for study design included: larger sample population with biopsy-confirmed absence of rejection, more rigorous classification, inclusion of important and frequent confounding conditions and prospective in-place validation under everyday clinical conditions to determine the practical value of non-invasive tests.  Recommendations for Endpoints included: emphasis on early markers which can detect subclinical stages of rejection, development of markers which can indicate response to the rejection therapy and prospective, randomized studies to determine the costs and benefits.  Recommendations for Technical aspects included: Uniform sample collection, preparation and analyses, development of simplified test systems which can be applied outside highly specialized laboratories, reliable measures for the test system and identification of confounders that reduce the sensitivity or specificity. |  |
| Han (2022)  China [21] | Review Article |  |  |  |  | 25 differentially expressed proteins (DEPs) overlapped in proteomic studies of urine and blood samples. Analysis showed that the DEPs  were mainly involved in the immune system and blood coagulation. Pathway analysis  showed that the complement and coagulation cascade pathways were well enriched.  Immunoglobulin heavy constant alpha 1 (IGHA1) and immunoglobulin k constant (IGKC) showed good performance in distinguishing AR from non-rejection groups validated with several datasets |  |
| Heidari (2021)  Iran [22] | Case control Study | 36 KTR, 12 with AMR 14 with stable graft function.  Validation for epidermal growth factor (19 AR, 18 without AR and 12 healthy volunteers without transplant) | Detection of AMR | Urine sample collected prior to biopsy | Label  Free  Quantification  (LFQ)  Proteomics. ELISA for validity. | 20 differentially expressed proteins with the highest sensitivity and specificity and combination of EGF, COL6A, and NID-1 was identified as possible panel for early diagnosis of AMR. Applicability of EGF as diagnostic biomarker was validated in urine samples of  independent set of AMR subjects. | Proteomics data showed elevation of some proteins that their biological significance in AMR process have not been yet understood |
| Herath (2019)  Australia [23] | Review Article |  |  |  |  | Difficulty of proteomics advancing as a field due to difficulty comparing to “gold-standard” diagnostics |  |
| Heyne (2012) Germany [24] | Case Control | n=182  Group 1 – Stable allograft function n=138  Group 2 – AKI due to biopsy proven TMCR n= 9  Group 3 – Post transplant AKI of other cause n=35 | Differentiating TCMR from other causes of AKI post-transplant | Urine | ELISA | urinary Neutrophil Gelatinase-Associated Liopcalin (NGAL) to allow for differential diagnosis of AKI, accurately predicting acute allograft rejection as underlying pathology  highest urinary NGAL concentrations were seen in those patients subsequently diagnosed with allograft rejection by histopathology, compared with AKI of all other cause. | Classification of AKI based on an increase in serum creatinine concentration, so identified only at an advanced stage of AKI or allograft rejection. |
| Ho (2016)  Canada [25] | Retrospective observational study | Normal histology (n = 5), IFTA (n = 6), subclinical (n = 6) and clinical rejection (n = 6). Larger prospective cohort n=133 | Detection of clinical and subclinical IFTA | Urine | LC-MS/MS analysis. Validated with ELISA. | Urinary MMP7: Cr and CXCL10: Cr significantly distinguished noninflamed from inflamed biopsies (area under the curve, 0.74 and 0.70, respectively). The addition of urinary MMP7: Cr to CXCL10: Cr improved the diagnostic  performance for subclinical and clinical inflammation/injury by integrated discrimination improvement (P= 0.002) and net reclassification improvement (P=0.006) analyses. | Incomplete histological reporting of samples which may have contaminated different rejection phenotype groups. |
| Ho (2017)  Canada [26] | Review Article |  |  |  |  | Future work requires external validation studies. Functional proteomic studies rather than simple quantitative proteomic work has potential to better understand kidney transplant pathophysiology and identify future biomarkers |  |
| Hricik (2013) USA [27] | Prospective multicentre observational trial | n=282  280 were kidney transplant recipients including 40 children,192 recipients of living donor allografts and 80 African Americans. | Detection of AR | Urine | SELDI-TOF-MS and ELISA | Mean values for CXCL10 protein were similar in patients with AR and infection, but values for CXCL9 protein were higher in patients with AR versus those with infection or other diagnoses  low urinary CXCL9 protein could be used to rule out AR, the data in this trial suggest that measurements of urinary CXCL9 protein are better than the urinary mRNAs tested for this purpose  low 6-month urinary CXCL9protein identifies patients without subclinical allograft injury and who are most likely to maintain stable kidney function | No self-reported limitations |
| Hussien (2020) Egypt [28] | Case control | 75 participants: Group I: 25 patients with IFTA, Group II: 25 transplanted patients with stable renal functions, and  Group III: 25 healthy control subjects | Diagnosis of IFTA | Urine | MALDI-TOF MS. | identified a proteomic peak pattern for IFTA. 5 peaks represented the proteomic profile that differentiates between the IFTA patients and the control with sensitivity of 100%, specificity of 100%, recognition capability of 100%, and cross-validation of 91.7% and five peaks differentiate between the transplant patients with normal renal functions and the control groups with sensitivity of 96.8%, specificity of 95.5%, recognition capability of 98%, and cross-validation of 100% | No self-reported limitations |
| Jacobs-Cachá (2017)  Spain [29] | Case Control Study | Group 1 – Kidney transplanted patients n=27  Group 2 – Healthy volunteers n=13  Group 3 – non-transplanted CKD patients n=9 | Levels of fascin-1 in CNIT | Urine and serum | ELISA | urinary but not serum (results not shown) fascin-1 levels were statistically elevated in CNI-treated kidney-transplanted patients exhibiting isometric vacuolization (histological hallmark for CNI toxicity), in comparison to healthy, CKD patients or CNI-treated kidney-transplanted patients without IV  - raised fascin-1 not seen in the CKD cohort  -fascin-1 translocation from cytosol to the extracellular media, points towards a potential effect of CNIs modulating fascin-1 function and location So,fascin-1 may represent a non-invasive urinary biomarker to assess tubular damage induced by CNIs. | further clinical assays shall be validated in more extensive cohorts |
| Jahnukainen (2006)  America [30] | Case Control Study | Group1 - 21 patients with BKVAN,  Group 2 -28 patients  with AR (Banff Ia to IIb),  Group 3-29 patients with stable graft function. | Differentiation of BK virus associated allograft nephropathy from acute allograft rejection based on proteomic peaks | Urine and serum | SELDI Mass Spectrometry | Able to detect several peaks that were differentially expressed in the BKVAN group compared with both the AR and stable function groups based on the urine samples  most significant peaks(m/z ratio [chip type]) that differentiated BKVAN samples  from the other two groups were 5.872 (CM10, IMAC30), 11.311  (CM10, IMAC30), 11.929 (CM10), 12.727 (CM10), and 13.349(CM10, IMAC30) kD peaks, most significant being 11.311  Normally, SELDI MS TOF has poor reproducibility but not in this study | Further studies on a larger number of patients needed to validate the identity of the significantly different peaks to develop robust, non-invasive methods for BKVAN diagnostic |
| Jeon (2022) South Korea [31] | Cohort Study | 50 Kidney Transplant Recipients | Renal Allograft Function- no specific condition | Urine | Liquid chromatography-mass spectrometry and tandem mass spectrometry  Validated by ELISA | levels of urinary RBP4/creatinine were inversely correlated with allograft function in KTRs  urinary RBP4 was higher in the rapid renal function decline group than in the stable renal function group | -small sample -single centre study -relatively short-term follow- up period. -Histologic diagnoses through kidney biopsy were not per- formed in all KTRs -Serum levels of RBP4 were not measured |
| Jia (2009) China [32] | Case Control | Group 1 – Acute clinical rejection n=10  Group 2 – Stable transplant n=10 | Detection of acute rejection | Urine | MALDI-TOF-  MS/MS and Western Blot | by the comparison of urine samples between  before and after renal graft AR, the alteration of AACT, GP96 and ZAG in urine was found to be significantly related to the AR  Clinical validation proved that AACT, GP96 and ZAG could significantly change 3 days earlier than the standard clinical criteria | Western Blot not the most accurate quantitative method , ELISA and larger cohort recommended |
| Jin (2022) America [33] | Review |  |  |  |  | Future clinical trials using the assays discussed in this review will further refine the performance of these assays, particularly the PPV and NPV values  Another area of focus is to identify biomarkers to distinguish TCMR from AMR because so far  neither blood nor urine-based markers provide a clear distinction |  |
| Johnston (2011) Ireland [34] | Retrospective case control study | Group 1 –34 renal transplant patients with histologically  proven IFTA Group 2 -36 patients with normal renal transplant function | Detection of biopsy proven IFTA | Urine | SELDI-TOF-MS validated by ELISA | Different protein peaks in both the control and the IFTA patient samples. Ten-fold increase in b2 microglobulin levels between patients with normal graft function and patients with IFTA is statistically relevant. | -protocol renal transplant biopsies were not carried out and therefore the number of IFTA patients in the study with mild IFTA was very small -more control groups were not included, e.g. patients with urinary tract infections -A larger cohort of patients would be required to confirm whether b2 microglobulin is specific for IFTA |
| Jung (2020) South Korea [35] | Cross-sectional multi-centre | 385 KTRs  - 26 KTRs with biopsy proven IFTA  - 57 KTRs with Long term graft survival  - 10 KTRs with rejection free | Detection of IFTA | Urinary vesicles | label-free liquid chromatography with tandem mass spectrometry and Western Blot Analysis | Identified and validated six proteomic biomarkers of IFTA and clarified one IFTA-specific proteomic biomarker in KTR AZGP1 was significantly increased in the CAMR group compared to the level in the control group APOA1 was the  most effective at distinguishing CAMR from LGS | -protocol biopsy was not performed in KTRs with LGS - potential proteomic biomarkers for CAMR may just reflect the rejection process itself |
| Kaisar (2019) United Kingdom [36] | Preliminary pilot study | 38 brain-dead kidney donors and matched recipients, 2 groups formed based on recipient’s graft outcome  Good outcome n=19  Suboptimal outcome n=19 | Kidney transplant outcomes based on 3 and 12 month post-transplant eGFR. No specific condition. | Donor kidney biopsy samples | Label-free quantification mass spectrometry and Western Blot | Different quantities of proteins in SO vs GO group:  In SO versus GO, significantly increased expression of the protein STAT-1. Increased TGF B1 in SO group. In GO versus SO, we were able  to observe enrichment of several cytoprotective proteins like thioredoxin-1,glutathione S-transferase | Small cohort used |
| Kanzelmeyer (2019) Germany [37] | Case control pilot study | 24 paediatric patients  with a diagnosis of IFTA  and 36 control patients (DSA-test  negative, normal kidney biopsy or no biopsy) | Detection of IFTA | Urine | Capillary Electrophoresis and Mass Spectrometry | Potential biomarker peptides were shown to be increased in IFTA group while others were decreased compared to control. 3 peptides (alpha-1-antitrypsin, annexin A1,neurosecretory protein VGF) showed a positive-fold change, whereas four peptides (beta-2-microglobulin, fibrinogen alpha, Ig kappa chain C region, retinol-binding protein 4) had a negative fold change. | A follow-up trial in a sufficiently large prospective cohort that will allow calculating more precise positive and negative predictive values of the combined proteomics test |
| Kienzl-Wagner (2013) USA [38] | Review Article |  |  |  |  | Major current hurdles in biomarker discovery in transplantation are  inconsistency in sample handling, processing and storage of bodily fluids such as blood, urine- the need for standardisation  The need for larger cohort and multi-centre studies |  |
| Kienzl-Wagner (2011) USA [39] | Review Article |  |  |  |  | robust study design with appropriate statistical power, blinding and validation is of crucial importance to improve reliability of proteomic-driven results. A panel of biomarkers has better diagnostic value than a single biomarker |  |
| Kurian (2009) USA [40] | Case control study  Multicentre | Kidney Transplant recipients with histology documented IFTA  n=77 | Detection of biopsy-proven IFTA | Serum | Liquid Chromatography Mass Spectrometry (LC/MS/MS) | Different proteomic profiles for the different stages of IFTA including 135 proteins unique to mild and 322 unique to moderate/severe IFTA | Limited use of results need a prospective clinical trial in  kidney transplantation with serial blood monitoring |
| Lepoittevin (2022) France [41] | Review Article |  |  |  |  | -The need for standardisation across studies  -increased availability of high-definition equipment in  hospital biochemistry services such as mass spectrometer has allowed for such advancements |  |
| Lim (2018) South Korea [42] | Cross-sectional multi-centre study | 2 groups  Group 1 -22  patients  with  stable  graft  function with no histological abnormality  Group- 2  25  Patients with  biopsy-proven  RMCR | Detection of TMCR | Urinary vesicles | nano-ultra  performance  liquid  chromatography-tandem  mass  spectrometry  (nano-UPLC-  MS/MS) and Western Blot Analysis | 46 proteins increased in Group 1  17 proteins increased in Group 2 i.e. those with TMCR  tetra-spanin-1 and  hemopexin were significantly  higher in TMCR patients  (STA:TMCR ratio=1:1.8,P=0.009,  And 1:3.5,P=0.046, respectively)  Other candidate biomarkers PIGR, APOA1, andLGALS3BP showed no significant difference | Small sample size  Insufficient study period |
| Lin (2015) China [43] | Case control | 2 groups n=19  Group 1- Patients with IFTA n=11  Group 2 – Control group of patients with renal cell carcinoma n=8 | Detection of IFTA | Kidney biopsy specimens | labelled with iTRAQ reagents  High performance liquid chromatography and tandem mass spectrometry  (HPLC-MS/MS) | 87 proteins showed significant differences in expression between the IFTA and control groups; 53 were up-regulated and 34 were down-regulated. | Need larger cohort and longer follow up time |
| Ling (2010) USA [44] | Cross sectional | 70 samples (50 from renal transplant, 20 healthy controls) | Acute rejection | Urine | Mass Spectrometry | Peptide sequencing revealed suggestive mechanisms of graft injury with roles for proteolytic degradation of uromodulin (UMOD) and several collagens, including COL1A2 and COL3A1. The 40-peptide panel discriminated AR in training (n =46) and test (n =24) sets (area under ROC curve =0.96). | Small sample |
| Loftheim (2012) Norway [45] | Case control study | 6 patients  with biopsy- proven acute rejection (AR) 6 age-matched controls without clinical signs of rejection | Detection of biopsy proven AR | Urine | Two-dimensional LC-MS/M | 11 proteins fulfilled predefined criteria for regulation in association with AR. They presented detectable  regulation already several days before clinical suspicion of AR (increased plasma creatinine).  Growth-related proteins (IGFBP7, Vasorin, EGF and Galectin-3-binding protein) were significantly up-  regulated in association with AR (P  = 0.03) while proteins related to immune response (MASP2, C3,CD59,Ceruloplasmin, PiGR and CD74) tended to be up-regulated (P= 0.13). | Small sample size  control group patients were not verified non-rejectors by protocol biopsies. |
| Mao (2007) China [46] | Case control study | n=73  Group 1 – stable graft function n=36  Group 2 – subclinical rejection n=37 | Diagnosis of subclinical rejection | Urine | SELDI-TOF-MS | The diagnostic model to differentiate stable recipients from SCR  group was comprised of 4 biomarkers with m/z of 2761, 10762, 11729  and 11940 Da. The peak of 2761 and 10762 were highly expressed in  stable group, while the other two peaks were highly expressed in SCR  group. | No self-reported limitations |
| Mas (2011) USA [47] | Review Article |  |  |  |  | Major challenge to this area is defining study end points and integrating these biomarkers into practice |  |
| McManus (2006) Ireland [48] | Review Article |  |  |  |  | A key focus should be the development of a panel of biomarkers as opposed to an individual protein peak  Studies confirm the use and convenience of urine as a sample |  |
| Menon (2016) USA and Australia [49] | Review Article |  |  |  |  | Multicentre prospective trials are required to validate these  candidate markers before meaningful conclusions can be made |  |
| Mertens (2020) Belgium [50] | Multicentre case-control study | Training cohort, n=249  Those with AMR - 60  Those without AMR = 189  validation n=391  Those with AMR - 43  Those without AMR -348 | Detection of antibody mediated rejection | Urine | nano–reversed-phase liquid chromatography and shotgun mass spectrometry | Identified a 10 protein panel that  reaches a very high negative predictive value (99%)  These were Alpha-1 B glycoprotein (A1BG); afamin (AFM); apolipoprotein A1 (APOA1); apolipoprotein A4 (APOA4); Ig heavy constant a1 (IGHA1); Ig heavy constant g4 (IGHA4); leucine rich a2 glycoprotein 1 (LRG1); alpha-1 anti- trypsin (SERPINA1); antithrombin (SERPINC1); and transferrin (TF). =  differed significantly between patients with versus without ABMR | -using mass spectrometry–based proteomics, the minimum set of proteins to make a model seems to consist of at least 6 proteins. However, more accessible quantification of these proteins could be considered–for example, using enzyme-linked immunoassay  -nonspecificity of the separate proteins in this urinary biomarker  - needs further evaluation in larger prospective studies with repeated sampling. |
| Metzger (2011) Germany [51] | Case control study | n=39  16 cases with  subclinical TMCR and 23 nonrejection controls | Detection of subclinical TMCR | Urine | Capillary electrophoresis mass spectrometry (CE-MS) | 387 peptides were identified to be significant but multivariate  statistical analysis identified only one peptide (ID-3796) with a  p-value of 0.05. With this single peptide, a sensitivity of 95% was  obtained, but specificity was only 61%. | Acute humoral  rejections were not included in the study  Small sample size |
| Mezzolla (2021) Italy [52] | Review Article |  |  |  |  | Translation of proteomic biomarkers into clinical practice is inhibited by lack of standardisation of study design.  Emphasis on panel of biomarkers rather than a specific protein |  |
| Moser (2017) Canada [53] | Observation Study | N=41  Kidney donors – including living donors and circulatory or brain dead donors | Delayed graft function based on creatinine measurement. Delayed graft function (DGF) | Perfusion Solution | Two dimensional gel electrophoresis  Liquid chromatography-Mass Spectrometry (LC-MS)  Western blot analysis | 7 protein spots were significantly  different between the 3 groups- these were identified as serum albumin, A1AT, peroxiredoxin 2, heavy chain of immunoglobulin, fragment of collagen 1, fatty acid binding protein (FABP), and protein deglycase (DJ-1) | the levels of proteins in the perfusate could reflect the levels at which the proteins are synthesized, actively released, or even passively released after an injury or all of the above reasons |
| Mortensen (2020) Denmark [54] | Observation Study | 31 renal transplant patients | IFTA | Kidney graft biopsies | Nano-LC-MSMS combined with 10-plex tandem mass tags (TMT) | Proteins- Coagulation Factor XIII A chain, Actin-related protein 2/3 subunit 2,Cytochrome C Oxidase Assembly Factor 6 homolog and Uridine Phosphorylase 1= showed a strong correlation with renal allograft fibrosis. | Need a larger cohort to validate the prognostic value of identified proteins  Including perivascular  fibrosis served to minimize the bias introduced by subjective evaluation, but may have caused the  overestimation of the extent of fibrosis, and thus weakened the correlation to renal function |
| Nakorchevsky (2010)  Canada [55] |  | 32 KTR with varying levels of Banff classification for IFTA | IFTA | Renal transplant biopsy | Tandem Mass Spectrometry | Multiple sets of proteins were mapped to different functional pathways, many increasing with histologic severity, including immune responses, inflammatory cell activation, and apoptosis consistent with the chronic rejection hypothesis. Two examples include the extensive population of the alternative rather than the classical complement pathway, previously not appreciated for IFTA, and a comprehensive control network for the actin cytoskeleton and cell signalling of the acute-phase response. |  |
| Navarrete (2019) Canada [56] | Review Article |  |  |  |  | Urine may not be the most ideal sample due to the potential changes due to pH alterations.  Even if an enzyme does not prove to be a useful biomarker, it helps evolve current knowledge in the field and can further highlight gaps in the research. |  |
| Nickerson (2009)  USA [57] | Review Article |  |  |  |  | Previous research, such as Quintana et al, whilst useful, requires validation in larger cohorts |  |
| Ong (2015)  USA [58] | Review Article |  |  |  |  | Substantial progress has been made in the use of proteomics in renal transplant medicine but current identified biomarkers require more robust validation in more complex transplant recipient populations. |  |
| O’Riordan (2005)  USA [59] | Cross-sectional Study | Biopsy-proven acute rejection (n=23) compared with trans-  plant recipients with stable graft function (  n=22) and healthy  volunteers (n=20) | Detection of AR | Urine at routine follow-up or time of biopsy for AR | SELDI-TOF mass spectrometry | Patients with AR could be distinguished from stable patients (sensitivity of 90.5-91.3% and specificity of 77.2- 83.3%). Protein masses that were important in constructing the classification algorithms included those of mass 2.0, 2.8, 4.8, 5.9, 6.9, 19.0, and 25.7 kDa. Normal urine was distinguished from transplant urine using a protein marker of mass 78.5 kDa with both a  sensitivity and a specificity of 100%. | Need to chemically define peaks. Small sample size. |
| O’Riordan (2007) USA [60] | Cross-sectional study | 31 stable transplant graft recipients and 42 with acute rejection | Detection of AR | Urine | SEDI-TOF-MS | The ratio of the 4.7 kDa to 4.4 kDa peptide’s peak intensities was significantly different in stable versus acute rejection groups  Ratio of b-Defensin-1and a-1-antichymotrypsin excretion in the urine is a novel, potentially useful candidate biomarkers of acute rejection | require further validation in a prospective biopsy-controlled trial |
| O’Riordan (2008)  UK [61] | Cross-sectional Study | 75 renal transplant recipients and 20 healthy volunteers | Detection of IFTA | Urine | Surface enhanced laser desorption and ionization MS | Patients could be classified into subgroups with normal histology and Banff CAN grades 2-3 with a  sensitivity of 86% and a specificity of 92% by applying the classification algorithm Adaboost to  urinary proteomic data. Several urinary proteins associated with advanced CAN were identified  including α1-micro-globulin, β2-micro-globulin, prealbumin, and endorepellin, the antiangiogenic  C-terminal fragment of perlecan. Increased urinary endorepellin was confirmed by ELISA and increased tissue expression of the endorepellin/perlecan ratio by immunofluoresence analysis of renal biopsies | not all urine specimens on which proteomic analysis was performed subsequently had  endorepellin assayed by ELISA due to shortage of clinical material |
| Peng (2008)  China [62] | Observational study | 132 kidney transplant recipients with stable graft function, 80 healthy controls | Subclinical rejection | Serum and urine at time of protocol biopsy.2 months post-transplant. | ELISA | Levels of VEGF in serum (126.96 ± 20.13 pg/mL;  95% confidence interval [95% CI], 83.10-170.83) and urine (16.14 ± 4.09 ng/mmol creatinine; 95% CI, 7.21-25.06) of 13 patients with subclinical rejection significantly differed from those of 119 patients with no allograft rejection (No-AR) and health controls.  The areas under the ROC curve were 0.771 (95% CI, 0.0.64-0.901) and 0.819 (95% CI, 0.662-0.976), respectively. Levels of VEGF in serum and urine after antirejection therapy (50.45 ± 6.58 pg/mL and 2.60 ± 0.83 ng/mmol creatinine, respectively) were lower than those at the time of protocol biopsy. No difference in urinary and serum VEGF expression was observed between cyclosporine and tacrolimus treatment. | Short follow-up time and small amount of subclinical rejection |
| Perez (2016)  Brazil [63] | Case control Study | 31 samples, including 6 from patients with AR and 25 from KTR with stable renal graft function | Detecting AR | Serum | LC-MS/MS | As results nineteen proteins were upregulated in the rejection group compared to the control group, and two proteins were downregulated: and three  were present exclusively in the rejection group. After analysis, we selected four proteins that were related to the acute phase response and that were strongly associated with each other: they are alpha-1 antitrypsin (A1AT), alpha-2 antiplasmin (A2AP), serum amyloid A (SAA) and apolipoprotein CIII (APOC3) |  |
| Pianta (2015) Australia [64] | single-centre, prospective cohort study | renal transplant recipients (N=81)  33 had immediate graft function  25 had slow graft function  23 had delayed graft function | Allograft function based on creatinine | Urine | ELISA | At 4 hr, receiver operating characteristic analysis suggested that urinary clusterin, IL-18, kidney injury molecule-1, and NGAL concentration were predictive of DGF.  Both urinary clusterin and IL-18 are useful biomarkers and may allow triaging of patients with DGF within 4 hr of transplantation. | modest size of the cohort and the absence of an independent validation cohort. A comprehensive risk model requires a much larger data and external validation |
| Pisitkun (2012)  USA [65] | Observational study | 18 KTR (7 ATN, 6 TMCR, 3 AMR and 2 stable function) | Detecting rejection or tubular injury | Urine exosomal at time of biopsy | LC-MS/MS | More than 1000 proteins were identified in each pathologic group. These protein lists were analysed computationally to identify Biological Process and KEGG Pathway terms that are significantly associated with each pathological group. Among the most informative terms for each group were: “sodium ion transport” for tubular injury; “immune response” for all rejection; “epithelial cell differentiation” for cell-mediated rejection; and “acute inflammatory response” for antibody-mediated rejection | Small sample number |
| Prunotto (2011)  Italy [66] | Review Article |  |  |  |  | Information derived from purely proteomic studies are limited but remains a promising area of research that has the potential to be incorporated clinically. |  |
| Quintana (2009)  Spain [67] | Cohort study | 71 individuals were included in the present study: an  initial training experiment analysed 18 patients with clinical and his-  to pathological characterization of IFTA and 14 controls  2^nd^ Validation group | IFTA | Urine | Label-free quantitative  LC-MS | Although unsupervised hierarchical clustering differentiated between the groups when including all the identified peptides, specific peptides derived from uromodulin and kininogen were found to be significantly more abundant in control than in IFTA patients and correctly identified the two groups. These peptides are therefore potential biomarkers that might be used for the diagnosis of IFTA. | Small sample numbers |
| Quintana (2009)  Spain [68] | Proof of concept Study | 32 patients with IFTA (14 with pure interstitial fibrosis and tubular atrophy and 18 with chronic active antibody-mediated rejection) and 18 control subjects (eight stable recipients and 10 healthy control subjects) | Detection of graft dysfunction | Urine pre-biopsy | MALDI-Mass Spectrometry | Unsupervised hierarchical clustering showed good segregation of samples in groups corresponding mainly to the four biomedical conditions. Moreover, the composition of the proteome of the pure interstitial fibrosis and tubular atrophy group differed from that of the chronic active antibody-mediated rejection group, and an independent validation set confirmed these results. The 14 protein ions that best discriminated between these two groups correctly identified 100% of the patients with pure interstitial fibrosis and tubular atrophy and 100% of the patients with chronic active antibody-mediated rejection. | Additional studies including a larger and more diverse transplant recipient population are required to confirm data. |
| Ramalhete (2022)  Portugal [69] | Review |  |  |  |  | It is therefore to develop standardized protocols for sample processing (e.g., whole blood samples), further evaluate protein quantification, to conduct better study designs while including a higher dimension population with a corresponding diversity of pathophysiological states. It is also paramount to evaluate patients throughout time and include independent and large-scale validation processes. |  |
| Rambabova-Bushljetik (2021) N. Macedonia [70] | Proof of concept study | 52 live donor recipients | Composite outcome of graft loss or death (8 year follow-up) | Urine at 24 months | CE-MS | CKD273 showed significant correlation with serum creatinine at every time point and moderate inverse correlation for the slope in glomerular filtration rates by Nankivell (r = −0.29, P = 0.05). Receiver operating characteristics analysis for graft loss and death within the next 6 years after proteomic analysis resulted in an area under curve value of 0.89 for CKD273 being superior to 0.67 for Nankivell eGFR. Stratification into CKD273-positive and -negative patient groups revealed a hazard ratio of 16.5 for prevalence of graft loss in case of CKD273 positivity | Strict phenotyping of patients, small numbers |
| Reichelt (2005) Germany [71] | Case control | 23 patients (13 with biopsy proven IFTA and 10 with no rejection on biopsy) | IFTA discrimination | Urine samples | Mass Spectrometry | Several protein peaks were identified allowing differentiation between rejection and no rejection. Using two different ProteinChip surfaces, we found two biomarkers at 25.71 kDa and 28.13 kDa that gave a diagnostic sensitivity of 90% and 93% and a specificity of 80% (SAX2) and 85% (CM10), respectively. | Small sample numbers |
| Rotondi (2009) Italy [72] | Retrospective case control study | 252 kidney transplant recipients separated into 2 groups based on their pre-transplant CXCL9 levels  (<272.1 pg/ml vs.>272.1 pg/ml)  Healthy controls n=50 | Detection of AR of transplant | Serum (pre-transplant stored) | ELISA | mean serum levels of CXCL9 were significantly higher in adult kidney graft recipients vs healthy subjects  Patients with normally functioning grafts showed significantly lower pretransplant serum CXCL9 levels than those patients who experienced graft failure throughout a 5-year follow-up  pretransplant level of circulating CXCL9 greater than 272.1 pg/ml and the occurrence of AR had a significant predictive power for allograft loss. |  |
| Schaub  (2004)  Canada [73] | Observational study | Acute clinical rejection group (n = 18), stable transplant group (n = 22), acute tubular necrosis group (n = 5), and recurrent (or de novo) glomerulopathy group (n = 5). Urines collected the day of the allograft biopsy were analysed by mass spectrometry. As a normal control group, 28 urines from healthy individuals were analysed the identical manner, as well as 5 urines from non-transplanted patients with lower urinary tract infection | AR of transplant | Urine  (Taken at time of biopsy) | Mass spectrometry | Three prominent peak clusters were found in 17 of 18 patients (94%) with acute rejection episodes, but only in 4 of 22 patients (18%) without clinical and histologic evidence for rejection and in 0 of 28 normal controls (P < 0.001). In addition, the presence or absence of these peak clusters correlated with the clinicopathologic course in most patients. Acute tubular necrosis, glomerulopathies, lower urinary tract infection, and cytomegalovirus viremia were not confounding variables. |  |
| Schaub (2005)  Canada [74] | Case control study | 63 | Acute tubular injury | Urine (stored) | LC-MALDI-MS/MS & SELDI-TOF-MS | Identification of these proteins peaks by mass spectrometry demonstrated that they all derive from non-tryptic cleaved forms of b2-microglobulin. In vitro experiments showed that cleavage of intact b2-microglobulin requires a urine pH <6 and the presence of aspartic proteases. Patients with acute tubulointerstitial rejection had lower urine pH than stable transplants and healthy individuals. In addition, they had higher amounts of aspartic proteases and  Intact b2-microglobulin in urine. These factors ultimately lead to increased amounts of cleaved urinary b2-microglobulin. | Antibodies to cleaved b2-microglubluin are unavailable limiting use of ELISA which would be more clinically applicable. |
| Schaub (2009) Canada [75] | Cohort | n=88  Group 1 – normal histology n=24  Group 2 – subclinical borderline tubulitis n=15  Group 3 – subclinical tubilitis Ia/b n=22  Group 4 – Clinical Tubulitis Ia/b n=17  Group 5 – IF/TA n=10 | Detection of subclinical tubulitis | Urine | ELISA | Urinary CXCL9 and CXCL10 concentrations demonstrated a close correlation with the extent of subclinical tubulitis, while no such distinction was seen for urinary CXCL4, CXCL11, CCL2and tubular injury markers. This supports an important role of CXCL9 and CXCL10 in the early rejection process | day-to-day variances of chemokine excretion need to be established for reliable interpretation of a single measurement. |
| Sigdel (2010)  USA [76] | Observational study | 92 KTR samples with AR, stable graft function, proteinuria and healthy controls.  *Children and Young adults | Detection of AR | Urine | Shotgun proteomics applying  LC-MS/MS and ELISA | Specific urinary proteins in AR, primarily relating to MHC antigens, the complement cascade and extra-cellular matrix proteins. A subset of proteins (UMOD, SERPINF1 and CD44), have been further cross validated by ELISA in an independent set of urine samples, for significant differences in the abundance of these urinary proteins in AR. | Difficult to interpret to adult population |
| Sigdel (2011)  USA [77] | Review Article |  |  |  |  | The lack of widely accepted protocols, difficulty in sample processing and transportation and a lack of collaborative efforts to achieve significant sample sizes in clinical studies has limited progress of biomarker discovery and clinical utilisation. |  |
| Sigdel (2014)  USA [78] | Observational study | 262 KTR urine samples. AR, stable graft, IFTA, CNIT, proteinuria from native renal disease and healthy control  Children and young adults. | Detection of renal transplant pathology | Urine | iTRAQ and LC-MS. ELISA for quantification. | 389 proteins measured displayed differential  abundances the injury types (p<0.05) with a significant finding that SUMO2 (small ubiquitin-related modifier 2) was identified as a “hub”  protein for graft injury irrespective of causation. 69 urine proteins had differences in abundance (p  <0.01) in AR compared with stable graft, of which 12 proteins were upregulated in AR with a mean fold increase of 2.8. Nine urine proteins were highly specific for AR because of their significant differences (p<0.01; fold increase >1.5) from all other transplant categories (HLA class II protein HLA-DRB1, KRT14, HIST1H4B, FGG, ACTB, FGB, FGA, KRT7, DPP4). Increased levels of three  of these proteins, fibrinogen beta (FGB; p  0.04), fibrinogen gamma (FGG; p 0.03), and HLA DRB1 (p 0.003) were validated by ELISA in AR using an independent sample set. The fibrinogen proteins further segregated AR from BK virus nephritis (FGB p 0.03, FGG p 0.02), a finding that supports the utility of monitoring these urinary  proteins for the specific and sensitive non-invasive diagnosis of acute renal allograft rejection | May not be generalisable to whole population given age of participants |
| Sigdel (2015)  USA [79] | Pilot Study | 30 samples (10 from KTR with AR, 20 without AR) | Detection of AR | Urinary exosome from urine samples taken at point of biopsy | iTRAQ and LC-MS | 11 proteins, functionally involved in an inflammatory and stress response, were more abundant  in samples from patients with AR, three of which are exclusive to the exosomes fraction.  Exosome AR-specific biomarkers were also detected in whole urine, but since they were observed at significantly lower abundances in they were unable to determine between AR and not AR |  |
| Sigdel (2016)  USA [80] | Observational cohort study | Urine samples for 245 KTR. Diagnostic categories of acute rejection, IFTA, BK virus nephritis, and stable graft.  151 as independent validation cohort | Detection of AR, IFTA, BK nephritis from stable graft | Urine | TRAQ-based or label-free LC-MS | A minimal set of 35 proteins were identified  for their ability to segregate the 3 major transplant injury clinical groups, - comprising the final  panel of 11 urinary peptides for acute rejection (93% AUC), 12 urinary peptides for chronic  allograft nephropathy (99% AUC), and 12 urinary peptides for BK virus nephritis (83% AUC). | High cost and limited availability of technology in clinical practice, |
| Sigdel (2022)  USA [81] | Abstract | 2 propensity score-matched UCLA kidney transplant patients of which 31 were CMV positive and 31 were CMV negative | Detection of CMV Viraemia | Serum 3 and 12 months after transplant, with additional samples 1 week and 1 month after viremia | LC-MS | 241 plasma proteins were able to separate samples based on CMV viremia status and based on post-viremia time. Analysis of plasma proteins at the baseline pre-infection resulted in a set of 17 proteins whose levels were either increased (n=6) or decreased (n=11). The most significantly increased protein was Lysine Methyltransferase 2C (KMT2C) with 3.38 fold increase (p=0.05) in CMV+ve samples and most significantly decreased protein was Immunoglobulin Lambda Variable 7-43 (IGLV7-43) with 2.17 fold decrease (p=0.01). The significant proteins were enriched in plasminogen activation and blood coagulation pathways as the top two biological pathways. The protein profile of baseline samples of CMV +ve patients was compared with protein profiles of 1-week post-viremia samples which resulted in ten significantly changed proteins (p<0.05). Increased proteins at the time of CMV+ve viremia (1-week post-viremia) included Serpin Family A Member 12 (SERPINA12) with p-value 0.01 and 2.47 fold increase and Immunoglobulin Heavy Variable 3-72 (IGHV3-72) with p-value 0.02 and 1.65 fold increase. Transthyretin (TTR ) and Lysine Methyltransferase 2C (KMT2C). CMV-specific proteins were enriched with functions such as protein activation cascade (p=1.42E-06) and regulation of acute inflammatory response (p=0.0001). | Small sample number |
| Smith  (2013)  UK [82] | Observational study | 55 KTR in initial analysis. Validation cohort of 194 KTR. | Detection of delayed graft function in perioperative patients (DGF) | Serum | Label free Mass Spectrometry subsequently ELISA for quantification | Analysis of longitudinal samples from an initial  validation cohort of 55 patients confirmed that the ACY-1 level on day 1 or 2 was a moderate predictor of delayed graft function, similar to serum creatinine, complementing the strongest predictor cystatin C. A further validation cohort of 194 patients confirmed this association with area under ROC curves (95% CI) for day 1 serum (138 patients) of 0.74 (0.67–0.85) for ACY-1, 0.9 (0.84–0.95) for cystatin C, and 0.93 (0.88–0.97) for both combined. Significant differences in serum ACY-1 levels were apparent between delayed, slow, and immediate graft function. Analysis of long-term follow- up for 54 patients with delayed graft function showed a highly significant association between day 1 or 3 serum ACY-1 and dialysis-free survival, mainly associated with the donor–brain–dead transplant type. | External validation required |
| Snoeijs (2013)  Netherlands [83] | Observational cohort study | 18 Donor kidneys (6 DBD, 6 DCD- controlled, 6 DCD uncontrolled) | Graft function following transplant | Perfusion Fluid | MS/MS | Two unidentified protein spots were significantly up-  regulated, whereas one protein spot – identified as haptoglobin – was significantly down-regulated in the perfusate of ischaemic injured kidneys from DCD as compared with kidneys DBD who had  not suffered warm ischaemic injury. Furthermore, two protein spots were up regulated in kidneys that never functioned after transplantation, whereas one spot was up-regulated – identified as a-1-antitrypsin – in kidneys with delayed graft function. | Unidentified proteins. Haptoglobin result was unable to be validated. |
| Song (2020)  USA [84] | Case control | 5 T-Cell mediated rejection, 5 BK nephropathy, 5 stable graft | Detection of TMCR and BKN | Renal Transplant Biopsy | Liquid Chromatography with Tandem Mass Spectrometry | 740 BKN and 638 TCMR associated proteins are significantly changed  compared to stable graft specimens. Principal component analysis demonstrated good segregation of all three phenotypes investigated. Protein detection and quantitation are highly reproducible: replicate comparative analyses  demonstrated 71–84% overlap of detected protein | Small sample numbers |
| Spasovski (2021)  N. Macedonia [85] | Review Article |  |  |  |  | Urine proves to be an advantageous sample due to its accessibility yet ability to provide useful information and is a promising area for development.  No identified biomarkers have made it to clinical practice routinely.  Suggests an idea of a panel of biomarkers rather than one single biomarker. |  |
| Srivastava (2011)  USA [86] | Case control study | Acute rejection (10), IFTA (11), Acute rejection (10)  Healthy Donors (8) | Detection of rejection | Urine- taken at point of biopsy for those with rejection | Large scale antibody microarrays.  Qualify of the  signals using the high throughput Reverse Capture Protein Microarray platform | ANXA11, Integrin α3 and Integrin β3, and TNFαinitially identified by the antibody microarray platform were all qualified using Reverse Capture Protein Microarrays | Small sample numbers |
| Stubendorff (2014) Germany [87] | Case control study | n=116  Group 1 – biopsy proven acute rejection n=58  Group 2 – stable graft function n=58 | Detection of AR | Urine | SELDI-TOF-MS | 117 spots were significantly differently regulated: 55 proteins were higher in AR group and 62 proteins were higher in ST group. Top identified candidates were AAT, A1MG and Hp  The combination of the markers resulted in a sensitivity of 85 % and specificity of 80 % | No self-reported limitations |
| Sui (2010)  China [88] | Pilot study | Patients with biopsy-confirmed acute renal allograft rejection (n=12), IFTA (n=12), stable graft function (n=12) and also from healthy volunteers (n=13) | Detection of rejection | Serum taken at time of biopsy and diagnosis | Matrix-assisted laser desorption ionization time-of-flight mass spectrometry (MALDI-  TOF MS) | 18 differential peptide peaks were shown as potential biomarkers of acute renal allograft rejection, and 6 differential peptide peaks were selected as potential biomarkers of chronic rejection. | Small sample |
| Tetaz (2012)  France [89] | Observational study | 29 KTR 3 months post transplantation | Predictive of IFTA | Urine | SELDI-TOF | The biomarker demonstrating the highest diagnostic performance was a protein of 8860 Da that predicted IFTA with a sensitivity of 93% and a specificity of 65%. Moreover, combination of these biomarkers in two multivariate analyses improved  the diagnostic potential of IFTA. Relevance of these individual biomarkers and a decisional algorithm constituted of 3 proteins was confirmed in an independent cohort of patients with undetermined CAD status one-year post-transplant | Larger scale studies with biopsy proven chronic allograft nephropathy required. |
| Tinel (2020)  France [90] | Review Article |  |  |  |  | Promising results from proteomic studies however translation into clinical practice remains limited. There will be a requirement for prospective interventional trials in order for novel proteomic technology to be incorporated into diagnostic clinical pathways. |  |
| Van Balkom (2017)  Netherlands [91] | Case control study | Perfusion fluid collected from 56 deceased donor kidneys | Predict delayed graft function (DGF) | Perfusion fluid of DCD Kidneys | Validated multiplex immunoassay based on Luminex technology | Variables tested for their contribution to a prediction model included five proteins (leptin, periostin, GM-CSF, plasminogen activator inhibitor-1, and osteopontin) and two clinical parameters (recipient body mass index [BMI] and dialysis duration) that distinguished between IF and DGF in the discovery set. Stepwise multivariable logistic regression provided a prediction model on the basis of leptin and GM-CSF. Receiver operating characteristic analysis showed an area under the curve (AUC) of 0.87, and addition of recipient BMI generated a model with an AUC of 0.89, outperforming the Kidney Donor Risk Index and the DGF risk calculator, showing AUCs of 0.55 and 0.59, respectively. | Small sample numbers |
| Van der Zwan (2018)  Netherlands [92] | Pilot Study | 11 patients with TMCR and 9 patients without TMCR | Detection of TMCR | Serum | Proximity extension immunoassay | Five proteins (CD5, CD8A, NCR1, TNFRSF4, and  TNFRSF9) were expressed significantly higher in samples with TMCR compared with samples without TMCR (adjusted P-value, 0.014) and had a good predictive capacity for TMCR [area under  the curve in a receiver– operator curve ranged from 0.83 to 0.91 (P,0.014)]. These proteins are associated with CD8+ cytotoxic T-cell and NK cell functions. In prerejection samples, IFN-alpha was expressed at a significantly lower level samples of patients without rejection. | No validation step |
| Van Leeuwen (2021)  Netherlands [93] | Pilot Study | 22 DBD KTR were selected (11 with good outcome and 11 with poor outcome) | 1 year graft outcome | Perfusion fluid used in DBD | LC-MS/MS | Hierarchical clustering of the 100 most abundant proteins showed discrimination between grafts with a good and poor at T1. Elevated levels of proteins involved in classical complement cascades at both T1 and T2 and a reduced abundance of lipid metabolism at T1 and of cytoskeletal proteins at T2 in GO versus SO was observed. ATP-citrate synthase and fatty acid-binding protein 5  (T1) and immunoglobulin heavy variable 2-26 and desmoplakin (T2) showed 91% and 86% predictive values, respectively, for transplant outcome. | Validation with larger sample required. |
| Wang (2011)  China [94] | Observational study | Enrolled patients included 38 biopsy-proved acute rejection,10 acute tubular necrosis, 24 subclinical rejection and 29 stable control recipients verified by protocol biopsy. | Detection of allograft dysfunction | Serum- taken prior to biopsy | SELDI-TOF MS | Characteristic protein profile can  be detected in each renal allograft dysfunction group. AR patients were differentiated from stable patients with markers of 9.7, 4.9, 66.8, 8.6, 6.7, 9.3 and 44.7 kDa with high sensitivity and specificity. ATN can be clearly distinguished from AR and stable control. Sub-clinical rejection differentiated from stable control with markers of 9.2, 2.8, 8.5 kDa. The independent blind test yielded with high specificity and sensitivity for each group. | Small sample numbers |
| Wang (2022)  China [95] | Cross sectional study | 10 KTR with BK Viraemia initially.  66 KTR with BK and 66 KTR without BK. | Allograft dysfunction secondary to BK viraemia | Serum | Mass Spectrometry and ELISA | 12 differentially expressed proteins, and S100A8 and S100A9 were the top two upregulated proteins. Plasma S100A8/ A9 was upregulated in patients with BKV. Plasma S100A8/A9 with 1 month creatine increase (ρ=0.499, p =0.021) and negatively correlated with 1-month eGFR (ρ =−0.618, p =0.003). Using least absolute shrinkage and selection operator regression models S100A8/A9 was found to be an independent risk factor for allograft dysfunction in KTR with BKV | Combination with other biomarkers may improve accuracy, |
| Williams (2017)  USA [96] | Case control study | KTR 21 DGF,15 SGF (slow graft function), and 16 IGF patients. | Detection of delayed graft function in perioperative phase of transplant (DGF) | Urine | ELISA | Four proteins (C4BPA, IGSF8, SAMP, and Guanylin (GUC2A)) were identified that together distinguished DGF with a sensitivity of 77.4%, specificity of 82.6% and AUC of 0.891. | No addition to clinical data to the model |
| Wittke (2005)  Germany [97] | Case control study | 19 patients with different grades of subclinical or clinical acute rejection (BANFF Ia to IIb), 10 patients with  urinary tract infection and 29 patients without evidence of rejection or infection | Detection of AR | Urine | Capillary electrophoresis coupled to mass spectrometry (CE-MS) | A distinct urinary polypeptide pattern identified 16/17 cases of acute tubolointerstitial rejection, but  was absent in two cases of vascular rejection. UTI resulted in a different polypeptide pattern that allowed differentiation between infection  and AR in all cases. Potentially confounding variables such as acute tubular lesions, tubular atrophy, tubulointerstitial fibrosis, calcineurin inhibitor toxicity, proteinuria, haematuria, allograft function and different immunosuppressive regimens did not interfere with test results. Blinded analysis of samples with and without rejection showed correct diagnosis by CE-MS in the majority of cases. | Given sample size unable to determine sensitivity and specificity. |
| Wu (2011)  China [98] | Case control Study | Plasma from 13  KTR was collected, of which 5  were from patients with AR before the therapy for rejection and 8 were from those without AR. | Detection of AR | Serum | iTRAQ with LC-MS/MS analysis | Among the 179 proteins identified by using iTRAQ labelling and quantitative proteomic  technology, 66 proteins were at least 2-fold different between patients with or without AR. The results demonstrated that the dominant processes and responses were associated with inflammation and complement activation in AR. A number of transcription factors were identified in AR patients, including nuclear factor-κB, signal transducer and activator of transcription 1, signal transducer and activator of transcription 3. | Low numbers prevented stratification of AR groups. |
| Yang (2010)  China [99] | Observational Study | 101 KTR included in study. 36 with stable function. 10 with ATN. 55 with AR (25 with AMR). | Detection of AMR | Urine | Surface-enhanced laser desorption/  ionization time-of-flight mass spectrometry. (SELDI-TOF-MS) | The ATN group was differentiated from the stable group with a sensitivity and specificity of  100% (pattern 1). The stable group was  differentiated from the AR group with a  specificity of 86.4% and a sensitivity of  85.4% (pattern 2). The C4d– ACR subgroup  was differentiated from the C4d+ AHR  subgroup with a specificity and sensitivity  of 95% and 80%, respectively (pattern 3). | Not Banff classified pathology. |
| Zhang (2009)  USA [100] | Observational Study | 6 populations were studied (A–F). Population “A” consisted of 120 approved  kidney donors samples collected before nephrectomy. Population  “B” contained 129 patients with native  kidney disease who were being evaluated for transplant.  Population “C” contained 41  KTR with grafts and  had a protocol biopsy with no pathology.  Population “D” contained serial urine samples from six  healthy volunteers providing three to six urine samples over  time periods ranging from 3 months to 2 years (four males  and two females aged 25–62 years). Population “E” contained individuals who were referred to the clinic because of  elevated creatinine and had a biopsy performed on the same day (n148, creatinine 2.27  0.90; GFR  34.0 13.4; 21 AR;  90 chronic rejection [including some with AR]; and 50 no  rejection).  Population “F”  These samples were from individuals with elevated creatinine and were collected at least 280 days before the final clinical data report (n159) | Graft function and episodes of rejection | Urine | MALDI-TOF MS | Patients with acute rejection showed profiles that ranged from those of kidney donors to those of  advanced kidney disease. The range of patterns may be useful for analysis of transplant patients without complications and persons with developing kidney disease before or after transplant. Urine from individuals with healthy kidneys showed few components other than two ubiquitous saposin B glycoisoforms. Patients with kidney disease lacked saposin B and showed new components in two patterns: the most common contained B-2 microglobulin. Pattern 2 lacked  B-2 microglobulin but contained several degradation products of a-1 antitrypsin. Other pathologic components included urinary protein 1 (m/z 15,835), transthyretin (m/z13,880), and a component at m/z 13,350. | Limited longitudinal data |
| Zhang (2020)  China [101] | Case control study | 12 KTR in total. 3 with AR and 9 healthy controls | Detection of Acute rejection | Serum | iTRAQ Mass Spectrometry | Using a cut-off of Q<0.05 and a fold change of >1.2 for the variation in expression, 109 proteins were identified to be differentially expressed between the AR and control groups, 72 of which were upregulated and 37 were downregulated. Several proteins, including properdin, keratin1, lipoprotein(a) and vitamin d-binding protein, may have roles in the pathogenesis of AR | Small sample study |

**Abbreviations**

AKI- Acute kidney injury

AMR- Antibody mediated rejection

AR- Acute rejection

ATN- Acute tubular necrosis

BKN- BK virus associated nephropathy

BKV- BK virus- Human polyomarvirus 1

CKD- Chronic kidney disease

CNIT- Calcineurin inhibitor toxicity

DBD- Donors after Brain Death

DCD- Donors after Circulatory Death

DGF- Delayed graft function

DSA- Donor specific antibodies

eGFR- Estimated glomerular filtration rate

ESKD- End-stage kidney disease

IFTA- Interstitial fibrosis and tubular atrophy

kDa- Kilodalton

KTR- Kidney transplant recipient

LC-MS/MS- Liquid Chromatography-MS/MS

MALDI-TOF MS- Matrix-assisted laser desorption/ionisation-time of flight MS

MS- Mass spectrometry

SELDI-TOF MS- Surface Enhanced Laser Desorption Ionisation Time of Flight MS

TCMR- T-cell mediated rejection

**References**

1. Al-Nedawi K, Haas-Neill S, Gangji A, Ribic CM, Kapoor A, Margetts P. Circulating microvesicle protein is associated with renal transplant outcome. Transpl Immunol. 2019 Aug;55:101210. doi: 10.1016/j.trim.2019.06.002.
2. Argani H. New Markers for Transplant Rejection. Exp Clin Transplant. 2020 Jan;18(Suppl 1):1-9. doi: 10.6002/ect.TOND-TDTD2019.L6.
3. Bañón-Maneus E, Diekmann F, Carrascal M, Quintana LF, Moya-Rull D, Bescós M, Ramírez-Bajo MJ, Rovira J, Gutierrez-Dalmau A, Solé-González A, Abián J, Campistol JM. Two-dimensional difference gel electrophoresis urinary proteomic profile in the search of nonimmune chronic allograft dysfunction biomarkers. Transplantation. 2010 Mar 15;89(5):548-58. doi: 10.1097/TP.0b013e3181c690e3.
4. Blydt-Hansen TD, Gibson IW, Gao A, Dufault B, Ho J. Elevated urinary CXCL10-to-creatinine ratio is associated with subclinical and clinical rejection in pediatric renal transplantation. Transplantation. 2015 Apr;99(4):797-804. doi: 10.1097/TP.0000000000000419.
5. Bohra R, Klepacki J, Klawitter J, Klawitter J, Thurman JM, Christians U. Proteomics and metabolomics in renal transplantation-quo vadis? Transpl Int. 2013 Mar;26(3):225-41. doi: 10.1111/tri.12003.
6. Braun F, Rinschen M, Buchner D, Bohl K, Plagmann I, Bachurski D, Richard Späth M, Antczak P, Göbel H, Klein C, Lackmann JW, Kretz O, Puelles VG, Wahba R, Hallek M, Schermer B, Benzing T, Huber TB, Beyer A, Stippel D, Kurschat CE, Müller RU. The proteomic landscape of small urinary extracellular vesicles during kidney transplantation. J Extracell Vesicles. 2020 Oct;10(1):e12026. doi: 10.1002/jev2.12026.
7. Brouard S, Ashton-Chess J, Soulillou JP. Surrogate markers for the prediction of long-term outcome in transplantation: Nantes Actualité Transplantation (NAT) 2007 meeting report. Hum Immunol. 2008 Jan;69(1):2-8. doi: 10.1016/j.humimm.2007.11.004.
8. Cassidy H, Slyne J, O'Kelly P, Traynor C, Conlon PJ, Johnston O, Slattery C, Ryan MP, McMorrow T. Urinary biomarkers of chronic allograft nephropathy. Proteomics Clin Appl. 2015 Jun;9(5-6):574-85. doi: 10.1002/prca.201400200.
9. Carreras-Planella L, Cucchiari D, Cañas L, Juega J, Franquesa M, Bonet J, Revuelta I, Diekmann F, Taco O, Lauzurica R, Borràs FE. Urinary vitronectin identifies patients with high levels of fibrosis in kidney grafts. J Nephrol. 2021 Jun;34(3):861-874. doi: 10.1007/s40620-020-00886-y.
10. Carreras-Planella L, Juega J, Taco O, Cañas L, Franquesa M, Lauzurica R, Borràs FE. Proteomic Characterization of Urinary Extracellular Vesicles from Kidney-Transplanted Patients Treated with Calcineurin Inhibitors. Int J Mol Sci. 2020 Oct 14;21(20):7569. doi: 10.3390/ijms21207569.
11. Chakraborty A, Sarwal M. Protein biomarkers in renal transplantation. Expert Rev Proteomics. 2018 Jan;15(1):41-54. doi: 10.1080/14789450.2018.1396892.
12. Chowdhury P, Hernandez-Fuentes MP. Non-invasive biomarkers to guide management following renal transplantation: the need for a multiplatform approach. Curr Opin Organ Transplant. 2013 Feb;18(1):1-5. doi: 10.1097/MOT.0b013e32835c8025.
13. Christians U, Klawitter J, Klawitter J. Biomarkers in Transplantation--Proteomics and Metabolomics. Ther Drug Monit. 2016 Apr;38 Suppl 1(Suppl 1):S70-4. doi: 10.1097/FTD.0000000000000243.
14. Cibrik DM, Warner RL, Kommareddi M, Song P, Luan FL, Johnson KJ. Identification of a protein signature in renal allograft rejection. Proteomics Clin Appl. 2013 Dec;7(11-12):839-49. doi: 10.1002/prca.201200036.
15. Clarke W, Silverman BC, Zhang Z, Chan DW, Klein AS, Molmenti EP. Characterization of renal allograft rejection by urinary proteomic analysis. Ann Surg. 2003 May;237(5):660-4; discussion 664-5. doi: 10.1097/01.SLA.0000064293.57770.42.
16. Clotet-Freixas S, McEvoy CM, Batruch I, Pastrello C, Kotlyar M, Van JAD, Arambewela M, Boshart A, Farkona S, Niu Y, Li Y, Famure O, Bozovic A, Kulasingam V, Chen P, Kim SJ, Chan E, Moshkelgosha S, Rahman SA, Das J, Martinu T, Juvet S, Jurisica I, Chruscinski A, John R, Konvalinka A. Extracellular Matrix Injury of Kidney Allografts in Antibody-Mediated Rejection: A Proteomics Study. J Am Soc Nephrol. 2020 Nov;31(11):2705-2724. doi: 10.1681/ASN.2020030286.
17. Freue GV, Sasaki M, Meredith A, Günther OP, Bergman A, Takhar M, Mui A, Balshaw RF, Ng RT, Opushneva N, Hollander Z, Li G, Borchers CH, Wilson-McManus J, McManus BM, Keown PA, McMaster WR; Genome Canada Biomarkers in Transplantation Group. Proteomic signatures in plasma during early acute renal allograft rejection. Mol Cell Proteomics. 2010 Sep;9(9):1954-67. doi: 10.1074/mcp.M110.000554.
18. Gao Y, Wu K, Xu Y, Zhou H, He W, Zhang W, Cai L, Lin X, Fang Z, Luo Z, Guo H, Chen Z. Characterization of acute renal allograft rejection by human serum proteomic analysis. J Huazhong Univ Sci Technolog Med Sci. 2009 Oct;29(5):585-91. doi: 10.1007/s11596-009-0511-8.
19. Günther OP, Shin H, Ng RT, McMaster WR, McManus BM, Keown PA, Tebbutt SJ, Lê Cao KA. Novel multivariate methods for integration of genomics and proteomics data: applications in a kidney transplant rejection study. OMICS. 2014 Nov;18(11):682-95. doi: 10.1089/omi.2014.0062.
20. Gwinner W, Metzger J, Husi H, Marx D. Proteomics for rejection diagnosis in renal transplant patients: Where are we now? World J Transplant. 2016 Mar 24;6(1):28-41. doi: 10.5500/wjt.v6.i1.28.
21. Han S, Zhao W, Wang C, Wang Y, Song R, Haller H, Jiang H, Chen J. Preliminary Investigation of the Biomarkers of Acute Renal Transplant Rejection Using Integrated Proteomics Studies, Gene Expression Omnibus Datasets, and RNA Sequencing. Front Med (Lausanne). 2022 May 12;9:905464. doi: 10.3389/fmed.2022.905464
22. Heidari SS, Nafar M, Kalantari S, Tavilani H, Karimi J, Foster L, Moon KM, Khodadadi I. Urinary epidermal growth factor is a novel biomarker for early diagnosis of antibody mediated kidney allograft rejection: A urinary proteomics analysis. J Proteomics. 2021 May 30;240:104208. doi: 10.1016/j.jprot.2021.104208.
23. Herath S, Erlich J, Au AYM, Endre ZH. Advances in Detection of Kidney Transplant Injury. Mol Diagn Ther. 2019 Jun;23(3):333-351. doi: 10.1007/s40291-019-00396-z.
24. Heyne N, Kemmner S, Schneider C, Nadalin S, Königsrainer A, Häring HU. Urinary neutrophil gelatinase-associated lipocalin accurately detects acute allograft rejection among other causes of acute kidney injury in renal allograft recipients. Transplantation. 2012 Jun 27;93(12):1252-7.
25. Ho J, Rush DN, Krokhin O, Antonovici M, Gao A, Bestland J, Wiebe C, Hiebert B, Rigatto C, Gibson IW, Wilkins JA, Nickerson PW. Elevated Urinary Matrix Metalloproteinase-7 Detects Underlying Renal Allograft Inflammation and Injury. Transplantation. 2016 Mar;100(3):648-54. doi: 10.1097/TP.0000000000000867
26. Ho J, Hirt-Minkowski P, Wilkins JA. New developments in transplant proteomics. Curr Opin Nephrol Hypertens. 2017 May;26(3):229-234. doi: 10.1097/MNH.0000000000000319.
27. Hricik DE, Nickerson P, Formica RN, Poggio ED, Rush D, Newell KA, Goebel J, Gibson IW, Fairchild RL, Riggs M, Spain K, Ikle D, Bridges ND, Heeger PS; CTOT-01 consortium. Multicenter validation of urinary CXCL9 as a risk-stratifying biomarker for kidney transplant injury. Am J Transplant. 2013 Oct;13(10):2634-44. doi: 10.1111/ajt.12426.
28. Johnston O, Cassidy H, O'Connell S, O'Riordan A, Gallagher W, Maguire PB, Wynne K, Cagney G, Ryan MP, Conlon PJ, McMorrow T. Identification of β2-microglobulin as a urinary biomarker for chronic allograft nephropathy using proteomic methods. Proteomics Clin Appl. 2011 Aug;5(7-8):422-31. doi: 10.1002/prca.201000160.
29. Jacobs-Cachá C, Torres IB, López-Hellín J, Cantarell C, Azancot MA, Román A, Moreso F, Serón D, Meseguer A, Sarró E. Fascin-1 is released from proximal tubular cells in response to calcineurin inhibitors (CNIs) and correlates with isometric vacuolization in kidney transplanted patients. Am J Transl Res. 2017 Sep 15;9(9):4173-4183.
30. Jahnukainen T, Malehorn D, Sun M, Lyons-Weiler J, Bigbee W, Gupta G, Shapiro R, Randhawa PS, Pelikan R, Hauskrecht M, Vats A. Proteomic analysis of urine in kidney transplant patients with BK virus nephropathy. J Am Soc Nephrol. 2006 Nov;17(11):3248-56. doi: 10.1681/ASN.2006050437.
31. Jeon HJ, Shin DH, Oh J, Kee YK, Park JY, Ko K, Lee S. Urinary Retinol-Binding Protein 4 is Associated With Renal Function and Rapid Renal Function Decline in Kidney Transplant Recipients. Transplant Proc. 2022 Mar;54(2):362-366. doi: 10.1016/j.transproceed.2021.10.028.
32. Jia X, Gan C, Xiao K, He W, Zhang T, Huang C, Wu X, Luo G, Wang X, Hu J, Tan J, Zhang X, Larsen PM, Wu J. Detection of urinary biomarkers for early diagnosis of acute renal allograft rejection by proteomic analysis. Proteomics Clin Appl. 2009 Jun;3(6):694-704. doi: 10.1002/prca.200800209.
33. Jin PH, Sarwal RD, Sarwal MM. Urinary Biomarkers for Kidney Allograft Injury. Transplantation. 2022 Jul 1;106(7):1330-1338. doi: 10.1097/TP.0000000000004017.
34. Johnston O, Cassidy H, O'Connell S, O'Riordan A, Gallagher W, Maguire PB, Wynne K, Cagney G, Ryan MP, Conlon PJ, McMorrow T. Identification of β2-microglobulin as a urinary biomarker for chronic allograft nephropathy using proteomic methods. Proteomics Clin Appl. 2011 Aug;5(7-8):422-31. doi: 10.1002/prca.201000160.
35. Jung HY, Lee CH, Choi JY, Cho JH, Park SH, Kim YL, Moon PG, Baek MC, Berm Park J, Hoon Kim Y, Ha Chung B, Lee SH, Kim CD. Potential urinary extracellular vesicle protein biomarkers of chronic active antibody-mediated rejection in kidney transplant recipients. J Chromatogr B Analyt Technol Biomed Life Sci. 2020 Feb 1;1138:121958. doi: 10.1016/j.jchromb.2019.121958.
36. Kaisar M, van Dullemen L, Charles P, Akhtar ZM, Thézénas ML, Huang H, Klooster A, Watkins NA, Kessler B, Ploeg RJ. Subclinical Changes in Deceased Donor Kidney Proteomes Are Associated With 12-month Allograft Function Posttransplantation-A Preliminary Study. Transplantation. 2019 Feb;103(2):323-328. doi: 10.1097/TP.0000000000002358.
37. Kanzelmeyer NK, Zürbig P, Mischak H, Metzger J, Fichtner A, Ruszai KH, Seemann T, Hansen M, Wygoda S, Krupka K, Tönshoff B, Melk A, Pape L. Urinary proteomics to diagnose chronic active antibody-mediated rejection in pediatric kidney transplantation - a pilot study. Transpl Int. 2019 Jan;32(1):28-37. doi: 10.1111/tri.13363.
38. Kienzl-Wagner K, Pratschke J, Brandacher G. Biomarker discovery in transplantation--proteomic adventure or mission impossible? Clin Biochem. 2013 Apr;46(6):497-505. doi: 10.1016/j.clinbiochem.2012.10.010.
39. Kienzl-Wagner K, Pratschke J, Brandacher G. Proteomics--a blessing or a curse? Application of proteomics technology to transplant medicine. Transplantation. 2011 Sep 15;92(5):499-509. doi: 10.1097/TP.0b013e3182265358.
40. Kurian SM, Heilman R, Mondala TS, Nakorchevsky A, Hewel JA, Campbell D, Robison EH, Wang L, Lin W, Gaber L, Solez K, Shidban H, Mendez R, Schaffer RL, Fisher JS, Flechner SM, Head SR, Horvath S, Yates JR, Marsh CL, Salomon DR. Biomarkers for early and late stage chronic allograft nephropathy by proteogenomic profiling of peripheral blood. PLoS One. 2009 Jul 10;4(7):e6212. doi: 10.1371/journal.pone.0006212.
41. Lepoittevin M, Kerforne T, Pellerin L, Hauet T, Thuillier R. Molecular Markers of Kidney Transplantation Outcome: Current Omics Tools and Future Developments. Int J Mol Sci. 2022 Jun 5;23(11):6318. doi: 10.3390/ijms23116318.
42. Lim JH, Lee CH, Kim KY, Jung HY, Choi JY, Cho JH, Park SH, Kim YL, Baek MC, Park JB, Kim YH, Chung BH, Lee SH, Kim CD. Novel urinary exosomal biomarkers of acute T cell-mediated rejection in kidney transplant recipients: A cross-sectional study. PLoS One. 2018 Sep 18;13(9):e0204204. doi: 10.1371/journal.pone.0204204.
43. Lin XC, Sui WG, Qi SW, Tang DE, Cong S, Zou GM, Zhang Y, Li H, Chen WB, Cheng ZQ, Dai Y. Quantitative proteomic profiling of renal tissue in human chronic rejection biopsy samples after renal transplantation. Transplant Proc. 2015 Mar;47(2):323-31. doi: 10.1016/j.transproceed.2014.10.010.
44. Ling XB, Sigdel TK, Lau K, Ying L, Lau I, Schilling J, Sarwal MM. Integrative urinary peptidomics in renal transplantation identifies biomarkers for acute rejection. J Am Soc Nephrol. 2010 Apr;21(4):646-53. doi: 10.1681/ASN.2009080876.
45. Loftheim H, Midtvedt K, Hartmann A, Reisæter AV, Falck P, Holdaas H, Jenssen T, Reubsaet L, Asberg A. Urinary proteomic shotgun approach for identification of potential acute rejection biomarkers in renal transplant recipients. Transplant Res. 2012 Aug 31;1(1):9. doi: 10.1186/2047-1440-1-9.
46. Mao Y, Yu J, Chen J, Yang H, He Q, Shou Z, Wu J, Zheng S. Diagnosis of renal allograft subclinical rejection by urine protein fingerprint analysis. Transpl Immunol. 2008 Jan;18(3):255-9. doi: 10.1016/j.trim.2007.08.008.
47. Mas VR, Mueller TF, Archer KJ, Maluf DG. Identifying biomarkers as diagnostic tools in kidney transplantation. Expert Rev Mol Diagn. 2011 Mar;11(2):183-96. doi: 10.1586/erm.10.119.
48. McManus CA, Rose ML, Dunn MJ. Proteomics of transplant rejection. *Transplant Rev* 2006; 20: 195–207.
49. Menon MC, Keung KL, Murphy B, OʼConnell PJ. The Use of Genomics and Pathway Analysis in Our Understanding and Prediction of Clinical Renal Transplant Injury. Transplantation. 2016 Jul;100(7):1405-14. doi: 10.1097/TP.0000000000000943.
50. Mertens I, Willems H, Van Loon E, Schildermans K, Boonen K, Baggerman G, Valkenborg D, Gwinner W, Anglicheau D, Essig M, Marquet P, Naesens M. Urinary Protein Biomarker Panel for the Diagnosis of Antibody-Mediated Rejection in Kidney Transplant Recipients. Kidney Int Rep. 2020 Jun 29;5(9):1448-1458. doi: 10.1016/j.ekir.2020.06.018.
51. Metzger J, Chatzikyrkou C, Broecker V, Schiffer E, Jaensch L, Iphoefer A, Mengel M, Mullen W, Mischak H, Haller H, Gwinner W. Diagnosis of subclinical and clinical acute T-cell-mediated rejection in renal transplant patients by urinary proteome analysis. Proteomics Clin Appl. 2011 Jun;5(5-6):322-33. doi: 10.1002/prca.201000153.
52. Mezzolla V, Pontrelli P, Fiorentino M, Stasi A, Pesce F, Franzin R, Rascio F, Grandaliano G, Stallone G, Infante B, Gesualdo L, Castellano G. Emerging biomarkers of delayed graft function in kidney transplantation. Transplant Rev (Orlando). 2021 Dec;35(4):100629. doi: 10.1016/j.trre.2021.100629.
53. Moser MAJ, Sawicka K, Arcand S, O'Brien P, Luke P, Beck G, Sawicka J, Cohen A, Sawicki G. Proteomic Analysis of Perfusate from Machine Cold Perfusion of Transplant Kidneys: Insights Into Protection from Injury. Ann Transplant. 2017 Dec 8;22:730-739. doi: 10.12659/aot.905347.
54. Mortensen LA, Svane AM, Burton M, Bistrup C, Thiesson HC, Marcussen N, Beck HC. Proteomic Analysis of Renal Biomarkers of Kidney Allograft Fibrosis-A Study in Renal Transplant Patients. Int J Mol Sci. 2020 Mar 30;21(7):2371. doi: 10.3390/ijms21072371.
55. Nakorchevsky A, Hewel JA, Kurian SM, Mondala TS, Campbell D, Head SR, Marsh CL, Yates JR 3rd, Salomon DR. Molecular mechanisms of chronic kidney transplant rejection via large-scale proteogenomic analysis of tissue biopsies. J Am Soc Nephrol. 2010 Feb;21(2):362-73. doi: 10.1681/ASN.2009060628.
56. Navarrete M, Wilkins JA, Lao Y, Rush DN, Nickerson PW, Ho J. Activity-based Protein Profiling Approaches for Transplantation. Transplantation. 2019 Sep;103(9):1790-1798. doi: 10.1097/TP.0000000000002752.
57. Nickerson P, Heeger PS. Proteomic portrayal of transplant pathologies. J Am Soc Nephrol. 2009 Feb;20(2):236-8. doi: 10.1681/ASN.2008121243.
58. Ong S, Mannon RB. Genomic and proteomic fingerprints of acute rejection in peripheral blood and urine. Transplant Rev (Orlando). 2015 Apr;29(2):60-7. doi: 10.1016/j.trre.2014.12.003.
59. O'Riordan E, Orlova TN, Mei J J, Butt K, Chander PM, Rahman S, Mya M, Hu R, Momin J, Eng EW, Hampel DJ, Hartman B, Kretzler M, Delaney V, Goligorsky MS. Bioinformatic analysis of the urine proteome of acute allograft rejection. J Am Soc Nephrol. 2004 Dec;15(12):3240-8. doi: 10.1097/01.ASN.0000145241.83482.68.
60. O'Riordan E, Orlova TN, Podust VN, Chander PN, Yanagi S, Nakazato M, Hu R, Butt K, Delaney V, Goligorsky MS. Characterization of urinary peptide biomarkers of acute rejection in renal allografts. Am J Transplant. 2007 Apr;7(4):930-40. doi: 10.1111/j.1600-6143.2007.01733.x.
61. O'Riordan E, Orlova TN, Mendelev N, Patschan D, Kemp R, Chander PN, Hu R, Hao G, Gross SS, Iozzo RV, Delaney V, Goligorsky MS. Urinary proteomic analysis of chronic allograft nephropathy. Proteomics Clin Appl. 2008 Jul;2(7-8):1025-35. doi: 10.1002/prca.200780137.
62. Peng W, Chen J, Jiang Y, Shou Z, Chen Y, Wang H. Prediction of subclinical renal allograft rejection by vascular endothelial growth factor in serum and urine. J Nephrol. 2008 Jul-Aug;21(4):535-42.
63. Perez JD, Sakata MM, Colucci JA, Spinelli GA, Felipe CR, Carvalho VM, Cardozo KH, Medina-Pestana JO, Tedesco-Silva H Jr, Schor N, Casarini DE. Plasma proteomics for the assessment of acute renal transplant rejection. Life Sci. 2016 Aug 1;158:111-20. doi: 10.1016/j.lfs.2016.06.029.
64. Pianta TJ, Peake PW, Pickering JW, Kelleher M, Buckley NA, Endre ZH. Clusterin in kidney transplantation: novel biomarkers versus serum creatinine for early prediction of delayed graft function. Transplantation. 2015 Jan;99(1):171-9. doi: 10.1097/TP.0000000000000256.
65. Pisitkun T, Gandolfo MT, Das S, Knepper MA, Bagnasco SM. Application of systems biology principles to protein biomarker discovery: urinary exosomal proteome in renal transplantation. Proteomics Clin Appl. 2012 Jun;6(5-6):268-78. doi: 10.1002/prca.201100108.
66. Prunotto M, Ghiggeri G, Bruschi M, Gabbiani G, Lescuyer P, Hocher B, Chaykovska L, Berrera M, Moll S. Renal fibrosis and proteomics: current knowledge and still key open questions for proteomic investigation. J Proteomics. 2011 Sep 6;74(10):1855-70. doi: 10.1016/j.jprot.2011.05.031.
67. Quintana LF, Campistol JM, Alcolea MP, Bañon-Maneus E, Sol-González A, Cutillas PR. Application of label-free quantitative peptidomics for the identification of urinary biomarkers of kidney chronic allograft dysfunction. Mol Cell Proteomics. 2009 Jul;8(7):1658-73. doi: 10.1074/mcp.M900059-MCP200.
68. Quintana LF, Solé-Gonzalez A, Kalko SG, Bañon-Maneus E, Solé M, Diekmann F, Gutierrez-Dalmau A, Abian J, Campistol JM. Urine proteomics to detect biomarkers for chronic allograft dysfunction. J Am Soc Nephrol. 2009 Feb;20(2):428-35. doi: 10.1681/ASN.2007101137.
69. Ramalhete LM, Araújo R, Ferreira A, Calado CRC. Proteomics for Biomarker Discovery for Diagnosis and Prognosis of Kidney Transplantation Rejection. Proteomes. 2022 Jul 2;10(3):24. doi: 10.3390/proteomes10030024.
70. Rambabova-Bushljetik I, Metzger J, Siwy J, Dohcev S, Bushljetikj O, Filipce V, Trajceska L, Mischak H, Spasovski G. Association of the chronic kidney disease urinary proteomic predictor CKD273 with clinical risk factors of graft failure in kidney allograft recipients. Nephrol Dial Transplant. 2022 Sep 22;37(10):2014-2021. doi: 10.1093/ndt/gfab297.
71. Reichelt O, Müller J, von Eggeling F, Driesch D, Wunderlich H, Schubert J, Gröne HJ, Stein G, Ott U, Junker K. Prediction of renal allograft rejection by urinary protein analysis using ProteinChip Arrays (surface-enhanced laser desorption/ionization time-of-flight mass spectrometry). Urology. 2006 Mar;67(3):472-5. doi: 10.1016/j.urology.2005.09.038.
72. Rotondi M, Netti GS, Lazzeri E, Stallone G, Bertoni E, Chiovato L, Grandaliano G, Gesualdo L, Salvadori M, Schena FP, Romagnani P, Serio M. High pretransplant serum levels of CXCL9 are associated with increased risk of acute rejection and graft failure in kidney graft recipients. Transpl Int. 2010 May 1;23(5):465-75. doi: 10.1111/j.1432-2277.2009.01006.x.
73. Schaub S, Rush D, Wilkins J, Gibson IW, Weiler T, Sangster K, Nicolle L, Karpinski M, Jeffery J, Nickerson P. Proteomic-based detection of urine proteins associated with acute renal allograft rejection. J Am Soc Nephrol. 2004 Jan;15(1):219-27. doi: 10.1097/01.asn.0000101031.52826.be.
74. Schaub S, Wilkins JA, Antonovici M, Krokhin O, Weiler T, Rush D, Nickerson P. Proteomic-based identification of cleaved urinary beta2-microglobulin as a potential marker for acute tubular injury in renal allografts. Am J Transplant. 2005 Apr;5(4 Pt 1):729-38. doi: 10.1111/j.1600-6143.2005.00766.x.
75. Schaub S, Nickerson P, Rush D, Mayr M, Hess C, Golian M, Stefura W, Hayglass K. Urinary CXCL9 and CXCL10 levels correlate with the extent of subclinical tubulitis. Am J Transplant. 2009 Jun;9(6):1347-53. doi: 10.1111/j.1600-6143.2009.02645.x.
76. Sigdel TK, Kaushal A, Gritsenko M, Norbeck AD, Qian WJ, Xiao W, Camp DG 2nd, Smith RD, Sarwal MM. Shotgun proteomics identifies proteins specific for acute renal transplant rejection. Proteomics Clin Appl. 2010 Jan;4(1):32-47. doi: 10.1002/prca.200900124.
77. Sigdel TK, Lee S, Sarwal MM. Profiling the proteome in renal transplantation. Proteomics Clin Appl. 2011 Jun;5(5-6):269-80. doi: 10.1002/prca.201000117.
78. Sigdel TK, Salomonis N, Nicora CD, Ryu S, He J, Dinh V, Orton DJ, Moore RJ, Hsieh SC, Dai H, Thien-Vu M, Xiao W, Smith RD, Qian WJ, Camp DG 2nd, Sarwal MM. The identification of novel potential injury mechanisms and candidate biomarkers in renal allograft rejection by quantitative proteomics. Mol Cell Proteomics. 2014 Feb;13(2):621-31. doi: 10.1074/mcp.M113.030577.
79. Sigdel TK, Ng YW, Lee S, Nicora CD, Qian WJ, Smith RD, Camp DG 2nd, Sarwal MM. Perturbations in the urinary exosome in transplant rejection. Front Med (Lausanne). 2015 Jan 5;1:57. doi: 10.3389/fmed.2014.00057.
80. Sigdel TK, Gao Y, He J, Wang A, Nicora CD, Fillmore TL, Shi T, Webb-Robertson BJ, Smith RD, Qian WJ, Salvatierra O, Camp DG 2nd, Sarwal MM. Mining the human urine proteome for monitoring renal transplant injury. Kidney Int. 2016 Jun;89(6):1244-52. doi: 10.1016/j.kint.2015.12.049.
81. Sigdel TK, Boada P, Kerwin M, Rashmi P, Gjertson D, Rossetti M, Sur S, Munar D, Cimino J, Ahn R, Pickering H, Sen S, Parmar R, Fatou B, Steen H, Schaenman J, Bunnapradist S, Reed EF, Sarwal MM; CMV Systems Immunobiology Group. Plasma proteome perturbation for CMV DNAemia in kidney transplantation. PLoS One. 2023 May 19;18(5):e0285870. doi: 10.1371/journal.pone.0285870.
82. Welberry Smith MP, Zougman A, Cairns DA, Wilson M, Wind T, Wood SL, Thompson D, Messenger MP, Mooney A, Selby PJ, Lewington AJ, Banks RE. Serum aminoacylase-1 is a novel biomarker with potential prognostic utility for long-term outcome in patients with delayed graft function following renal transplantation. Kidney Int. 2013 Dec;84(6):1214-25. doi: 10.1038/ki.2013.200.
83. Snoeijs MG, Pulinx B, van Dieijen-Visser MP, Buurman WA, van Heurn LW, Wodzig WK. Characterization of the perfusate proteome of human donor kidneys. Ann Clin Biochem. 2013 Mar;50(Pt 2):140-6. doi: 10.1258/acb.2012.011144.
84. Song L, Fang F, Liu P, Zeng G, Liu H, Zhao Y, Xie X, Tseng G, Randhawa P, Xiao K. Quantitative Proteomics for Monitoring Renal Transplant Injury. Proteomics Clin Appl. 2020 Jul;14(4):e1900036. doi: 10.1002/prca.201900036.
85. Spasovski G, Rambabova-Bushljetik I, Trajceska L, Dohcev S, Stankov O, Stavridis S, Saidi S, Dimitrovski K, Popov Z. Urinary Proteomics in Kidney Transplantation. Pril (Makedon Akad Nauk Umet Odd Med Nauki). 2021 Dec 30;42(3):7-16. doi: 10.2478/prilozi-2021-0030.
86. Srivastava M, Eidelman O, Torosyan Y, Jozwik C, Mannon RB, Pollard HB. Elevated expression levels of ANXA11, integrins β3 and α3, and TNF-α contribute to a candidate proteomic signature in urine for kidney allograft rejection. Proteomics Clin Appl. 2011 Jun;5(5-6):311-21. doi: 10.1002/prca.201000109.
87. Stubendorff B, Finke S, Walter M, Kniemeyer O, von Eggeling F, Gruschwitz T, Steiner T, Ott U, Wolf G, Wunderlich H, Junker K. Urine protein profiling identified alpha-1-microglobulin and haptoglobin as biomarkers for early diagnosis of acute allograft rejection following kidney transplantation. World J Urol. 2014 Dec;32(6):1619-24. doi: 10.1007/s00345-014-1263-z.
88. Sui W, Huang L, Dai Y, Chen J, Yan Q, Huang H. Proteomic profiling of renal allograft rejection in serum using magnetic bead-based sample fractionation and MALDI-TOF MS. Clin Exp Med. 2010 Dec;10(4):259-68. doi: 10.1007/s10238-010-0094-5.
89. Tetaz R, Trocmé C, Roustit M, Pinel N, Bayle F, Toussaint B, Zaoui P. Predictive diagnostic of chronic allograft dysfunction using urinary proteomics analysis. Ann Transplant. 2012 Jul-Sep;17(3):52-60. doi: 10.12659/aot.883458.
90. Tinel C, Anglicheau D. Suivi du patient ayant reçu une transplantation rénale par les biomarqueurs urinaires : de l’innovation technologique au développement clinique [Urinary biomarkers in kidney transplant recipients: From technological innovations to clinical development]. Nephrol Ther. 2021 Apr;17S:S83-S87.. doi: 10.1016/j.nephro.2020.03.003.
91. van Balkom BWM, Gremmels H, Ooms LSS, Toorop RJ, Dor FJMF, de Jong OG, Michielsen LA, de Borst GJ, de Jager W, Abrahams AC, van Zuilen AD, Verhaar MC. Proteins in Preservation Fluid as Predictors of Delayed Graft Function in Kidneys from Donors after Circulatory Death. Clin J Am Soc Nephrol. 2017 May 8;12(5):817-824. doi: 10.2215/CJN.10701016.
92. van der Zwan M, Hesselink DA, Clahsen-van Groningen MC, Baan CC. Targeted Proteomic Analysis Detects Acute T Cell-Mediated Kidney Allograft Rejection in Belatacept-Treated Patients. Ther Drug Monit. 2019 Apr;41(2):243-248. doi: 10.1097/FTD.0000000000000587.
93. van Leeuwen LL, Spraakman NA, Brat A, Huang H, Thorne AM, Bonham S, van Balkom BWM, Ploeg RJ, Kessler BM, Leuvenink HGD. Proteomic analysis of machine perfusion solution from brain dead donor kidneys reveals that elevated complement, cytoskeleton and lipid metabolism proteins are associated with 1-year outcome. Transpl Int. 2021 Sep;34(9):1618-1629. doi: 10.1111/tri.13984.
94. Wang M, Jin Q, Tu H, Mao Y, Yu J, Chen Y, Shou Z, He Q, Wu J, Zheng S, Chen J. Detection of renal allograft dysfunction with characteristic protein fingerprint by serum proteomic analysis. Int Urol Nephrol. 2011 Dec;43(4):1009-17. doi: 10.1007/s11255-011-9962-5.
95. Wang S, Su M, Lin J, Zhang L, Li J, Tian Y, Qiu W. S100A8/A9, an Upregulated Host Factor in BK Virus Infection after Kidney Transplantation, Is Associated with Allograft Function Impairment. J Proteome Res. 2022 Oct 7;21(10):2356-2366. doi: 10.1021/acs.jproteome.2c00219.
96. Williams KR, Colangelo CM, Hou L, Chung L, Belcher JM, Abbott T, Hall IE, Zhao H, Cantley LG, Parikh CR. Use of a Targeted Urine Proteome Assay (TUPA) to identify protein biomarkers of delayed recovery after kidney transplant. Proteomics Clin Appl. 2017 Jul;11(7-8):10.1002/prca.201600132. doi: 10.1002/prca.201600132.
97. Wittke S, Haubitz M, Walden M, Rohde F, Schwarz A, Mengel M, Mischak H, Haller H, Gwinner W. Detection of acute tubulointerstitial rejection by proteomic analysis of urinary samples in renal transplant recipients. Am J Transplant. 2005 Oct;5(10):2479-88. doi: 10.1111/j.1600-6143.2005.01053.x.
98. Wu D, Zhu D, Xu M, Rong R, Tang Q, Wang X, Zhu T. Analysis of transcriptional factors and regulation networks in patients with acute renal allograft rejection. J Proteome Res. 2011 Jan 7;10(1):175-81. doi: 10.1021/pr100473w.
99. Yang H, Mao Y, Yu J, Chen J, He Q, Shou Z, Wu J, Chen Y, Zheng S. Diagnosis of c4d+ renal allograft acute humoral rejection by urine protein fingerprint analysis. J Int Med Res. 2010 Jan-Feb;38(1):176-86. doi: 10.1177/147323001003800120.
100. Zhang Y, Oetting WS, Harvey SB, Stone MD, Monkkonen T, Matas AJ, Cosio FG, Nelsestuen GL. Urinary Peptide patterns in native kidneys and kidney allografts. Transplantation. 2009 Jun 27;87(12):1807-13. doi: 10.1097/TP.0b013e3181a66595.
101. Zhang Y, Ou M, Lin H, Lai L, Chen H, Chen J, Sui W, Xue W, Zhang R, Gan Q, Tang D, Sun X, Dong J, Yan Q, Dai Y. Proteomic analysis of differentially expressed proteins in the serum of patients with acute renal allograft rejection using iTRAQ labelling technology. Mol Med Rep. 2020 Sep;22(3):2329-2341. doi: 10.3892/mmr.2020.11299.
